# The controllable of BODIPY dimers without installing blocking groups as both fluorescence and singlet oxygen generators

**DOI:** 10.1002/smo.20240023

**Published:** 2024-07-21

**Authors:** Jianfang Cao, Tianci Zhang, Xinyu Chen, Xue Ma, Jiangli Fan

**Affiliations:** ^1^ School of Chemical Engineering Ocean and Life Sciences Dalian University of Technology Panjin China; ^2^ State Key Laboratory of Fine Chemicals Dalian University of Technology Dalian China

**Keywords:** BODIPY dimers, density functional calculations, dual‐functioning photosensitizers, spin‐orbit coupling

## Abstract

We compared a range of BODIPY dimer derivatives without installing blocking groups by optimizing geometry structures and analyzing energies, frontier molecular orbitals, *C*hole&*C*ele map, electron density difference, spin‐orbit coupling (SOC) matrix and decay rate constants from excited states. The dihedral angles of the *β*‐*β*‐linked BODIPY dimer and the *α*‐*α*‐linked BODIPY dimer tend to flatten in the T_1_ state, which is detrimental to the occurrence of the intersystem crossing (ISC). Conversely, the dihedral angle of the *meso*‐*β*‐linked BODIPY dimer, the *meso*‐*meso*‐linked BODIPY dimer and *α*‐*γ*‐linked BODIPY dimer is within the range of 125°–143° in the T_1_ state, facilitating ISC and the generation of singlet oxygen. Notably, the transition from S_1_ to S_0_ involving lowest unoccupied molecular orbital to highest occupied molecular orbital with long‐wavelength emission and moderate oscillator strength underpins the remarkable long emission peaks observed experimentally for *α*‐*γ*‐linked BODIPY dimer. Moreover, the apparent SOC matrix enhances the ISC process, resulting in a respectable efficiency in generating singlet oxygen for this dimer. In *meso*‐*β*‐linked BODIPY, *meso*‐*meso*‐linked BODIPY, and *α*‐*γ*‐linked BODIPY, the S_1_→T_1_ process is characterized by a significant charge transfer, specifically transitioning from the ^1^CT state to the ^3^LE state, indicative of a spin‐orbit charge transfer ISC (SOCT‐ISC) mechanism. The ability to regulate the photosensitivity of BODIPY dimers by adjusting the dihedral angle between the two units in the T_1_ state unveils new avenues for designing high‐performance photosensitizers for both therapeutic and imaging applications.

## Graphical abstract

The dihedral angle between the two BODIPY units in the T_1_ state significantly influences intersystem crossing process in BODIPY dimers. The *α*‐*γ*‐linked BODIPY dimer exhibits notable charge transfer during the S_1_→T_1_ transition, corresponding to a transition from the ^1^CT to ^3^LE state, driven by the SOCT‐ISC mechanism. With large SOC, low energy gap E_s1t1_, and strong conjugation, the *α*‐*γ*‐linked BODIPY dimer can significantly promote the generation of singlet oxygen and long‐wavelength fluorescence.

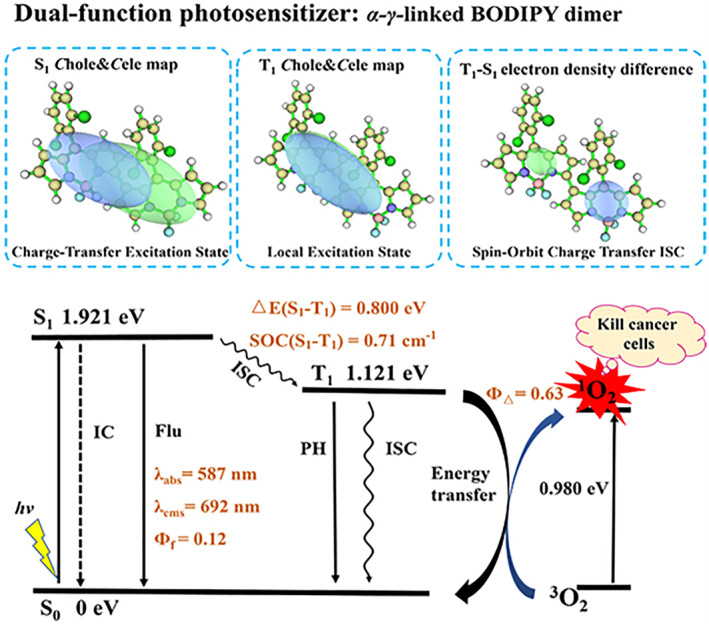

## 1 INTRODUCTION

As an emerging technology, the amalgamation of photoactivated cancer diagnosis and treatment has significant advantages in improving the treatment effect and reducing side effects of cancer. This integration is projected to expedite the advancement of cancer diagnostics technology.[^1^, ^2^, ^3^, ^4^, ^5^, ^6^] The energy from the light was absorbed by the sensitizer to induce the sensitizer to transfer from the ground state to the S_*n*_ (*n* ≥ 1) states. Then, photosensitizer molecules in singlet excited states can also reach T_*n*_ (*n* ≥ 1) states through intersystem crossing (ISC). In the T_1_ state, the photosensitizer molecules can react with the surrounding molecules or transfer energy to produce anticancer factors to treat the tumor. Fluorescence, internal conversion and the ISC are competitive processes, which significantly complicate the creation of photosensitizers that possess both high fluorescence quantum yield and the ability to produce substantial amounts of singlet oxygen.[^7^, ^8^, ^9^, ^10^, ^11^, ^12^, ^13^] These problems require molecular structural regulation to balance brightness and phototoxicity for tumor diagnosis and treatment.[^14^, ^15^, ^16^, ^17^, ^18^, ^19^, ^20^, ^21^, ^22^]

The molecular design of triplet photosensitizers based on the heavy atom effect can enhance the efficiency of the ISC process. However, incorporating heavy atoms into the molecular structure may lead to inherent defects such as short‐wavelength absorption, high cost, toxicity and relatively complex synthesis, which limitted the application of photosensitizers with heavy atoms in phototherapy diagnosis.[^23^, ^24^] Therefore, it is important to develop efficient triplet photosensitizers that are free of heavy atoms, which displayed high reactive oxygen species generation capacity, extended lifetime of the triplet state, good photostability and compatibility with biological systems, easy modification and low cost.[^25^, ^26^, ^27^, ^28^, ^29^, ^30^] BODIPY derivatives as heavy atom free photosensitizers have the ideal photosensitizer characteristics, excellent spectral characteristics and biocompatibility.[^31^, ^32^, ^33^, ^34^, ^35^, ^36^] Most heavy atom free BODIPY photosensitizers have mainly focused on promoting ISC.[^37^, ^38^, ^39^] Note that few photosensitizers have been developed that meet the requirements of possessing both high fluorescence emission and ISC rate capability. BODIPY‐phenoxazine, which was linked by a distyryl‐linked chain, exhibited strong capability in producing singlet oxygen and excellent fluorescence characteristics.^[38]^ The Hasobe group reported π‐extended BODIPY derivatives (Phena‐Mono‐BDP) with a high quantum yield of ISC (Φ_isc_ ≈ 0.63) and robust fluorescence quantum yield (Φ_f_ ≈ 0.56), which are suitable for photodynamic therapy (PDT) guided by fluorescence imaging.^[39]^ The development of dual‐functioning photosensitizers is still at an initial stage of research.

Direct‐linked BODIPY dimers are gradually attracting wide attention in PDT as heavy atom free photosensitizers, although only a small number of directly linked BODIPY dimers have been synthesized.[^40^, ^41^, ^42^, ^43^] The photosensitivity of the BODIPY dimer can be easily modulated by the dihedral angle between the two interconnected BODIPY units, due to different connection positions and steric hindrance at the 1,2,3,5,6,7‐positions (between the two linked BODIPYs). We proposed that BOP‐3‐3‐BOP, a constrained BODIPY dimer with methyl groups at the 1,3,5,7‐positions of the BODIPY core, demonstrates π‐conjugation between the two BODIPY units. Additionally, this compound features a narrow energy gap between its first singlet excited state (S_1_) and first triplet excited state (T_1_), coupled with an extensive spin‐orbit coupling (SOC) coefficient, which contributes to its substantial fluorescence efficiency and considerable singlet oxygen generation efficiency, rendering it advantageous for applications in fluorescence imaging and PDT.^[44]^

BODIPY dimers linked through *α*‐*α* and *α*‐*γ* bonds without installing blocking groups at the two, six‐positions were reported by Jiao. The BODIPY dimer linked through *α*‐*α* bond exhibited near infrared absorption wavelength and emission wavelength, strong fluorescence, but could not generate singlet oxygen. In contrast, *α*‐*γ* dimers with long absorption and emission can effectively generate singlet oxygen (*Φ*_Δ_≈0.63) combined with fluorescence quantum yield (*Φ*_f_ ≈ 0.12), which are promising for use as a dual‐functioning photosensitizer.^[45]^ Zhang reported that BOP‐8‐8‐BOP and d1, without steric hindrance at the 1,3,5,7‐positions, were superior photosensitizers for PDT, but were less fluorescent.[^46^, ^47^] Understanding the correlation between the structural characteristics and spectral properties of these BODIPY dimers by relying solely on experimental methods is challenging.

**Scheme 1:**
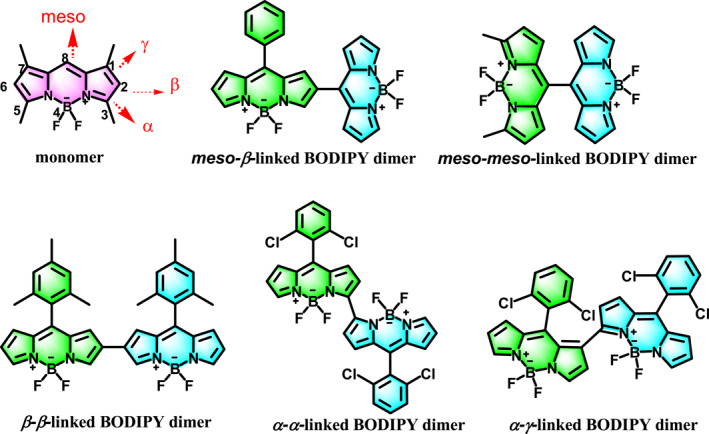
Chemical formulas of BODIPY dimers linked through *meso*‐*β*, *meso*‐*meso*, *β*‐*β*, *α*‐*α* and *α*‐*γ* bonds.

The comparison of the spectral characteristics of BODIPY dimers, specifically those without blocking groups, has never been systematically discussed. In the current work, a detailed theoretical investigation was carried out to reveal the mechanism behind the differences in optical properties and singlet oxygen yield, especially in comparison with BODIPY dimers linked through *meso*‐*β*,^[47]^ *meso*‐*meso*,^[46]^ *β*‐*β*,^[48]^ *α*‐*α*,^[45]^ and *α*‐*γ* ^[45]^ bonds that lack blocking groups (as shown in Scheme 1) documented in the literature.

Theoretical studies indicate that the different linkage positions between two BODIPY units affect the bond length and dihedral angle of the linkage for the BODIPY dimers without blocking groups. In addition, the different oscillator strengths of the transitions from the excited state back to the ground state explain the varying fluorescence quantum yields of BODIPY dimers. The *C*hole&*C*ele map, electron density difference (EDD), energy gap and SOC are modulated by different connection positions, which subsequently influence the efficiency of singlet oxygen generation. Even more, these studies may be beneficial for the design of BODIPY dimers without blocking groups and the development of high‐performance dual‐functioning photosensitizers.

## 2 COMPUTATIONAL APPROACH

We optimized the S_0_ state of these BODIPY dimers by density functional theory (DFT) and optimized the S_1_ state and T_1_ state of these BODIPY dimers by time‐dependent density functional theory (TD‐DFT). The adiabatic excitation energies and vertical excitation energies were calculated for the optimized the S_0_ state structures and the optimized the S_1_ state structures using the TD‐DFT method. APF‐D (Austin‐Petersson‐Frisch Dispersion) functional method and 6‐311+G (2d,p) basis set were suitable for BODIPY dimers.^[49]^

The APF‐D functional is a type of density functional used in computational chemistry. It is designed to include dispersion interactions, which are weak forces that play a crucial role in the stability and behavior of molecular systems. The inclusion of dispersion corrections helps improve the accuracy of calculations for non‐covalent interactions, such as van der Waals forces, which are often poorly represented in traditional density functionals.

6‐311+G (2d,p) basis set is part of the Pople basis sets, which are commonly used in quantum chemistry calculations. 6‐311+G (2d,p) basis set is a split‐valence triple‐zeta basis set. This means that the basis set includes three sets of basis functions for valence electrons (a triple‐zeta basis set), which allows for more flexibility and accuracy in representing the electron distribution. The “+” symbol signifies the inclusion of diffuse functions. Diffuse functions are particularly important for accurately modeling anions and excited states. The “(2days,p)” notation indicates the addition of polarization functions to the basis set. The “2days” means that two sets of d‐type polarization functions are added to heavy atoms (typically non‐hydrogen atoms), and the “p” refers to p‐type polarization functions added to hydrogen atoms. Polarization functions help to describe the angular variation in electron density, improving the accuracy of the basis set in capturing molecular geometries and bonding situations.

In addition, we used the solvation model density method based on the density solvation model to calculate solvent effects in dichloromethane solutions.^[50]^ We used the same method as geometric optimization to calculate the frequency analysis in order to find the true minimum of the structure. The potential energy curve of the ground state was obtained from a series of structures with restricted dihedral angles. The potential energy curve of the first excited state was calculated by the Frank‐Condon vertical transition energy on the basis of the ground state potential energy curve. Geometric optimization, energies and orbital analysis were performed for S_0_, S_1_ and T_1_ states using the Gaussian16 program suite.^[51]^ The Multiwfn 3.8 (dev) code was utilized to conduct *C*hole*&C*ele map, EDD.[^52^, ^53^] We calculated the SOC matrix using the sf‐X2C (scalar relativistic effects with spineless exact two‐component Hamiltonian) by Beijing Density Function (BDF) program.[^54^, ^55^, ^56^, ^57^, ^58^, ^59^, ^60^] The choice of functions for BDF was limited, and the SOC coefficients of atoms were calculated using the PBE0 function coupled with the cc‐pVDZ‐DK basis set. The rates of decay of molecular excited states were calculated using the molecular materials property prediction package program.[^61^, ^62^, ^63^, ^64^]

## 3 CALCULATION RESULTS AND ANALYSIS

### 3.1 Spectral characteristics of BODIPY dimers without installing blocking groups

The spectral data of BODIPY dimers without installing blocking groups collected in the literature are shown in Table 1. The *meso*‐*β*‐linked BODIPY dimer is connected at BODIPY positions *meso* and *β* without steric hindrance at the 1,3,5,7‐positions.^[47]^ The *meso*‐*β*‐linked BODIPY dimer without installing blocking groups, has *λ*_abs_ = 507 nm and *λ*_em_ = 514 nm in dichloromethane, low fluorescence quantum yields (0.08 in CH_2_Cl_2_) and high singlet oxygen quantum yields (0.24 in CH_2_Cl_2_), which is very close to the spectroscopic characteristics of conformation restricted *meso*‐*β*‐linked BODIPY dimer.^[40]^ We inferred that 1,3,5,7‐positions with or without methyl groups had little effect on the *meso*‐*β*‐linked BODIPY dimer spectrum by comparing the spectral properties of the two dyes.

**TABLE 1:** Photophysical characteristics of five types of BODIPY dimers with various linkages (*meso*‐*β*, *meso*‐*meso*, *β*‐*β*, *α*‐*α*, and *α*‐*γ*).

Dyes	*λ*_abs_ (nm)^a^	*λ*_em_(nm)^b^	Φ_f_ ^c^	Φ_Δ_ ^d^
*meso*‐*β*‐linked BODIPY dimer^[47]^	507^e^	514^e^	0.08^e^	0.24^f^
*meso*‐*meso*‐linked BODIPY dimer^[46]^	519^e^	53^e^	0.01^e^	0.87^f^
*β*‐*β*‐linked BODIPY dimer^[48]^	609^e^	655^e^	0.15^e^	‐‐
*α*‐*α*‐linked BODIPY dimer^[45]^	685^e^	725^e^	0.50^e^	‐‐
*α*‐*γ*‐linked BODIPY dimer^[45]^	587^e^	692^e^	0.12^e^	0.63^g^

^a^ Absorption maximum *λ*_abs_.

^b^ Emission maximum *λ*_em_.

^c^ Fluorescence quantum yields Φ_f_.

^d^ Singlet oxygen quantum yields Φ_Δ_.

^e^ In CH_2_Cl_2_.

^f^ In toluene.

^g^ Chloroform.

In dichloromethane, the *meso*‐*meso*‐linked BODIPY dimer, in which two BODIPY chromophores are directly connected at their *meso* positions without installing blocking groups at the 1,3,5,7‐positions, has an absorption wavelength *λ*_abs_ = 519 nm, an emission wavelength *λ*_em_ = 534 nm, a low fluorescence quantum yield of only 0.01, but a high singlet oxygen quantum yield of 0.87.^[46]^ In contrast, the restricted dimer *meso*‐*meso*‐linked BODIPY dimer has a moderate fluorescence quantum yield of 0.31 and a moderate singlet oxygen quantum yield of 0.46 in dichloromethane.^[40]^ We conclude that *meso*‐*meso*‐linked BODIPY dimer with non‐orthogonal configurations has a higher capability of generating singlet oxygen than the orthogonal configuration. However, the absence of restriction groups may lead to internal conversion, which in turn reduces the fluorescence efficiency.

The *β*‐*β*‐linked BODIPY dimer without steric hindrance at the 1,3,5,7‐positions showed a red‐shift of the absorption peak to 609 nm, as well as an emission peak at 655 nm. The fluorescence quantum yield was measured to be 0.15 in CHCl_3_, while no singlet oxygen quantum response was observed.^[48]^ In contrast, restricted dimer *β*‐*β*‐linked BODIPY dimer exhibited a blue‐shift in both the absorption and emission peaks, an apparent fluorescence response and a modest singlet oxygen response.^[42]^ By comparison, we found that the presence or absence of substituents at the 1,3,5,7‐positions significantly influenced the fluorescence quantum yield and singlet oxygen quantum yield of both *β*‐*β*‐linked BODIPY dimer and *meso*‐*meso*‐linked BODIPY dimer.

The *α*‐*α*‐linked BODIPY dimer without steric hindrance at the 2,6‐positions showed near‐infrared absorption and emission maxima at 685 and 725 nm, a fluorescence quantum yield of 0.50 in CH_2_Cl_2_ and no singlet oxygen response.^[45]^ In contrast, the restricted *α*‐*α*‐linked BODIPY dimer showed a shift towards higher energy in both absorption and emission peaks, a noteworthy quantum yield of fluorescence emission, and a moderate quantum yield of singlet oxygen.^[41]^

As an isomer of *α*‐*α*‐linked BODIPY dimer, the *α*‐*γ*‐linked BODIPY dimer, without steric hindrance at the 2,6‐positions, exhibited an absorption/emission maximum at 587/692 nm, a moderate fluorescence quantum yield of 0.12 (in CH_2_Cl_2_), and a high singlet oxygen quantum yield of 0.63 (in CHCl_3_).^[45]^ These characteristics make it a highly promising dual photosensitizer for use in cancer therapy. We hypothesize that the substantial disparities in the fluorescence and singlet oxygen quantum yields observed between the two isomers can be attributed to the conjugation effects and the energy level alignment between the S_1_ and T_1_ states. We further revealed the relationship between structure and property through computational chemistry.

### 3.2 Optimized molecular structures of BODIPY dimers without installing blocking groups in S_0_, S_1_ and T_1_ states

**FIGURE 1:**
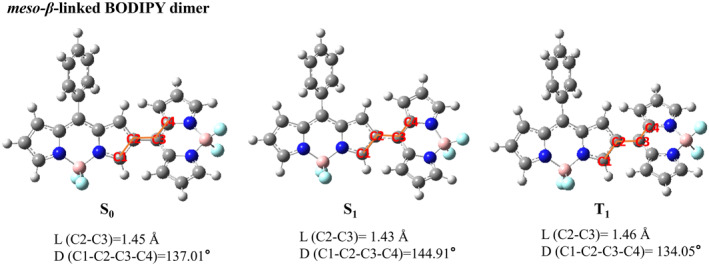
The optimized three‐dimensional geometric structures of *meso*‐*β*‐linked BODIPY dimer.

**FIGURE 2:**
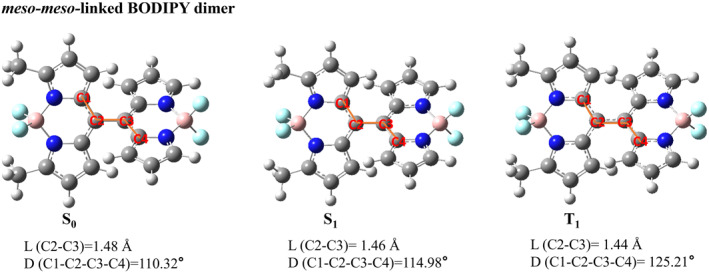
The optimized three‐dimensional geometric structures of *meso*‐*meso*‐linked BODIPY dimer.

**FIGURE 3:**
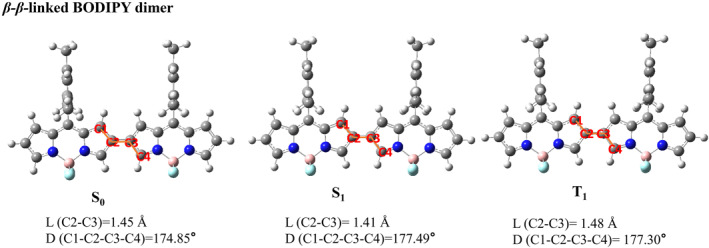
The optimized three‐dimensional geometric structures of *β*‐*β*‐linked BODIPY dimer.

**FIGURE 4:**
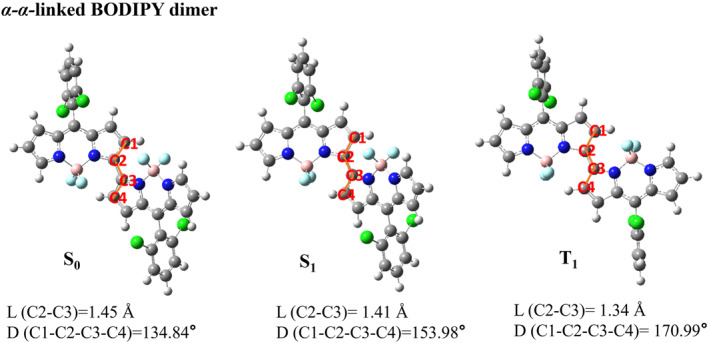
The optimized three‐dimensional geometric structures of *α*‐*α*‐linked BODIPY dimer.

**FIGURE 5:**
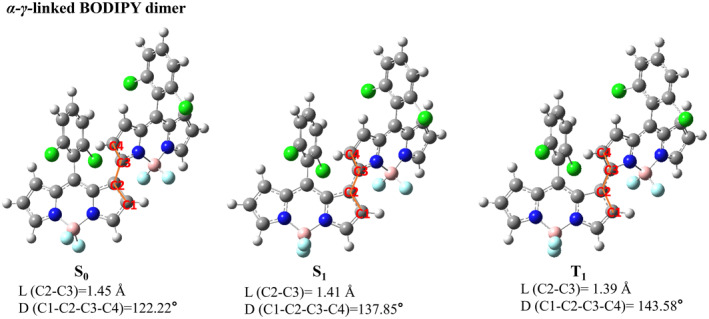
The optimized three‐dimensional geometric structures of *α*‐*γ*‐linked BODIPY dimer.

We presented the optimized three‐dimensional geometries of five types of BODIPY dimers in their ground state, the first excited singlet state, and the first excited triplet state. The main structural parameters of these five types of BODIPY dimers in S_0_, S_1_ and T_1_ states are listed in Figures 1, 2, 3, 4, 5.

It was found that the dihedral angle between two BODIPY units of *meso*‐*β*‐linked BODIPY dimer without installing blocking groups deviates from orthogonality towards planarization. The dihedral angles of *meso*‐*β*‐linked BODIPY dimer between the two BODIPY units are 137.01° in the S_0_ state and 144.91° in the S_1_ state, respectively, indicating that the S_1_ state could slightly flatten out after configuration relaxation. Additionally, in the S_1_ state, the bond length between the two BODIPY units is slightly reduced compared to that in the S_0_ state. The dihedral angles of the *meso*‐*meso*‐linked BODIPY dimer between the two BODIPY units were 110.32°, 114.98° and 125.21° in the S_0_, S_1_ and T_1_ states, respectively. The dihedral angles of *meso*‐*meso*‐linked BODIPY dimer without steric hindrance are less deviated from the vertical angle than those of the *meso*‐*β*‐linked BODIPY dimer. In the S_1_ and T_1_ state, the bond length between the two units is slightly shorter than that in the S_0_ state.

The *β*‐*β*‐linked BODIPY dimer has dihedral angles of 174.85°in the S_0_ state, 177.49° in the S_1_ state and 177.30° in the T_1_ state between the two BODIPY units, respectively, indicating that they are almost completely coplanar. The bond length between the two BODIPY individuals of the *β*‐*β*‐linked BODIPY dimer is 1.41 Å in the S_1_ state,which is similar to that of a π‐conjugated bond. The *α*‐*α*‐linked BODIPY dimer has a dihedral angle −134.84° between two BODIPY individuals in the S_0_ state, deviating from a vertical configuration. The BODIPY dihedral angle in the S_1_ state is −153.98°, indicating that the two BODIPY units are closer to coplanarity after configuration relaxation. In the T_1_ state,the *α*‐*α*‐linked BODIPY dimer has a dihedral angle of −170.99° between two BODIPY units. The bond length between two BODIPY individuals of the *α*‐*α*‐linked BODIPY dimer is 1.41 Å in the S_1_ state, inferring that there is a large conjugation between two BODIPY units. In the T_1_ state, *α*‐*α*‐linked BODIPY dimer and *β*‐*β*‐linked BODIPY dimer have the dihedral angles that tend to be planar between the two BODIPY units, which are not conducive to spin‐orbit charge transfer ISC.

The dihedral angles of *α*‐*γ*‐linked BODIPY dimer without installing blocking groups between two BODIPY individuals are −122.22° in S_0_ state, −137.85° in S_1_ state and −143.58° in T_1_ state respectively, which indicates stronger steric hindrance between the two BODIPY units than *α*‐*α*‐linked BODIPY dimer isomer. In the *α*‐*γ*‐linked BODIPY dimer, the bond length between two BODIPY units is 1.41 Å in the S_1_ state, akin to that of a π‐conjugated bond, ensuring strong fluorescent emission. At the same time, the *α*‐*γ*‐linked BODIPY dimer is non‐coplanar between the two BODIPY units and exhibits a certain degree of distortion. We speculate that this distorted structure not only facilitates charge separation during the S_0_→S_1_ transition but also promotes electron‐hole recombination during the S_1_→T_1_ transition. This will facilitate the transition from the ^1^CT state to the ^3^LE state driven by the SOCT‐ISC mechanism, followed by energy transfer to generate singlet oxygen. We will further verify this process using hole‐electron analysis and EDD.

### 3.3 The analysis of frontier orbital for BODIPY dimers without installing blocking groups

We used the TD‐DFT method to caculate the absorption and emission spectra, as well as the frontier orbitals of BODIPY dimers. Table S1 and S2 presented absorption wavelengths (*λ*_abs_ in nm) and emission wavelengths (*λ*_em_ in nm) of electron excited states with low energy levels for dimeric BODIPY dyes that were linked in various ways, including *meso*‐*β*, *meso*‐*meso*, *β*‐*β*, *α*‐*α*, and *α*‐*γ* linkages.

**FIGURE 6:**
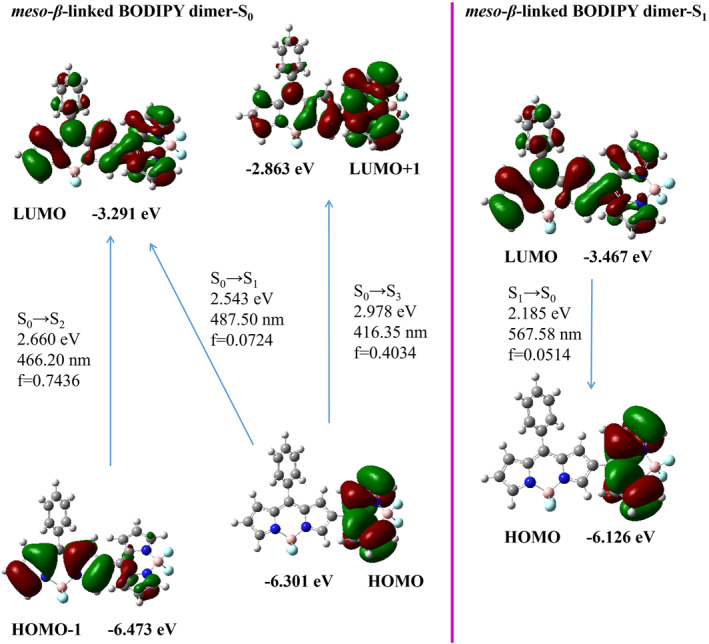
Frontier molecular orbitals of the optimized S_0_ state and the S_1_ state geometries for the *meso*‐*β*‐linked BODIPY dimer.

**FIGURE 7:**
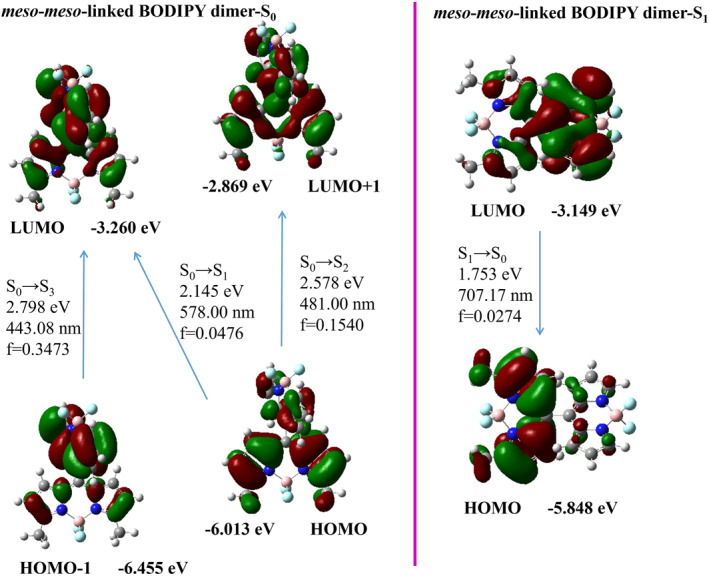
Frontier molecular orbitals of the optimized S_0_ state and the S_1_ state geometries for the *meso*‐*meso*‐linked BODIPY dimer.

The analysis of the frontier orbitals of the *meso*‐*β*‐linked BODIPY dimer in the S_0_ state revealed that the transitions from HOMO‐1 to lowest unoccupied molecular orbital (LUMO) and from highest occupied molecular orbital (HOMO) to LUMO+1 are analogous to those observed in the HOMO to LUMO transition of the BODIPY monomer (Figure 6). The overlap of molecular orbitals in the S_1_→S_0_ transition (LUMO→HOMO) and the oscillator strength of the electron transition (*f* = 0.0514) are very small, and we speculate that the S_1_→S_0_ transition (LUMO→HOMO) is a weak transition leading to weak fluorescence. The *meso*‐*meso*‐linked BODIPY dimer displayed HOMO‐1 to LUMO and HOMO to LUMO+1 orbital transitions in the frontier molecular orbitals of the S_0_ state that were akin to those observed in the HOMO to LUMO transition of the BODIPY monomer. The overlap of molecular orbitals in the S_1_→S_0_ transition (LUMO→HOMO) and the oscillator strength of the electron transition (*f* = 0.0274) are very small, and we speculate that the S_1_→S_0_ transition (LUMO→HOMO) is a weak transition that leads to weak fluorescence in Figure 7. Spectral and photophysical data revealed some analogies between the *meso*‐*meso*‐linked BODIPY dimer and *meso*‐*β*‐linked BODIPY dimer.

**FIGURE 8:**
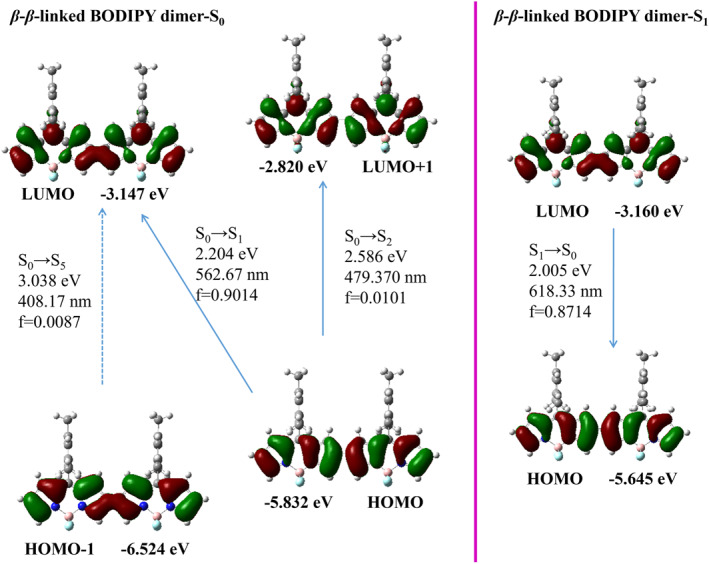
Frontier molecular orbitals of the optimized S_0_ state and the S_1_ state geometries for the *β*‐*β*‐linked BODIPY dimer.

**FIGURE 9:**
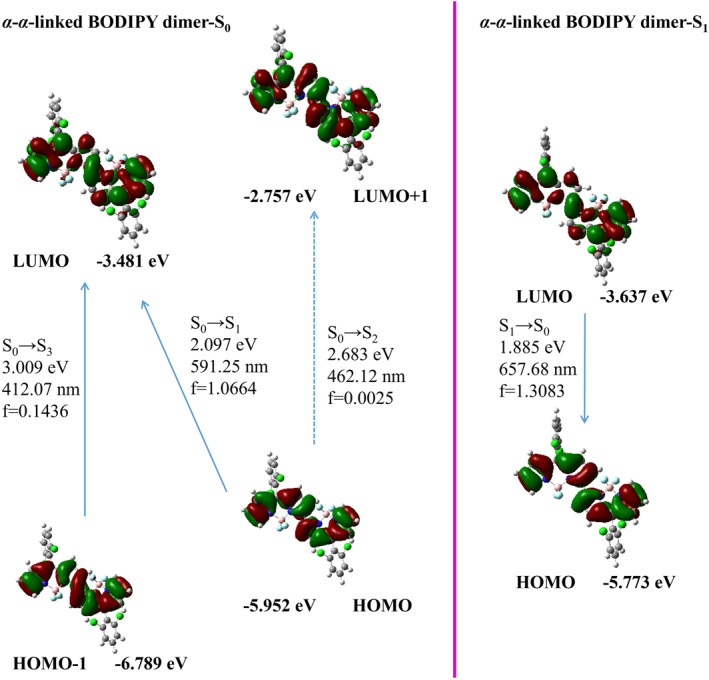
Frontier molecular orbitals of the optimized S_0_ state and the S_1_ state geometries for the *α*‐*α*‐linked BODIPY dimer.

In Figure 8, it can be observed that the *β*‐*β*‐linked BODIPY dimer's frontier molecular orbital in the S_0_ state underwent HOMO to LUMO transition with an oscillator strength of *f* = 0.9014. The *β*‐*β*‐linked BODIPY dimer exhibited a redshifted maximum emission peak (618.33 nm, *f* = 0.8714) in its excited state (S_1_) in contrast to the BOP‐Mon spectrum. This shift is indicative of the presence of π conjugation between the BODIPY units. The almost planar structure of the *β*‐*β*‐linked BODIPY dimer increased the degree of electron conjugation between two BODIPY units. The *α*‐*α*‐linked BODIPY dimer exhibited a main absorption band HOMO→LUMO transition (591.25 nm,  = 1.0664) under S_0_→S_1_ excitation, indicating that there was a great conjugate between the two BODIPY units (Figure 9). The transition of S_1_→S_0_ (657.68 nm, *f* = 1.3083, LUMO→HOMO) of *α*‐*α*‐‐‐linked BODIPY dimer resulted in a strong emission peak with a long wavelength.

**FIGURE 10:**
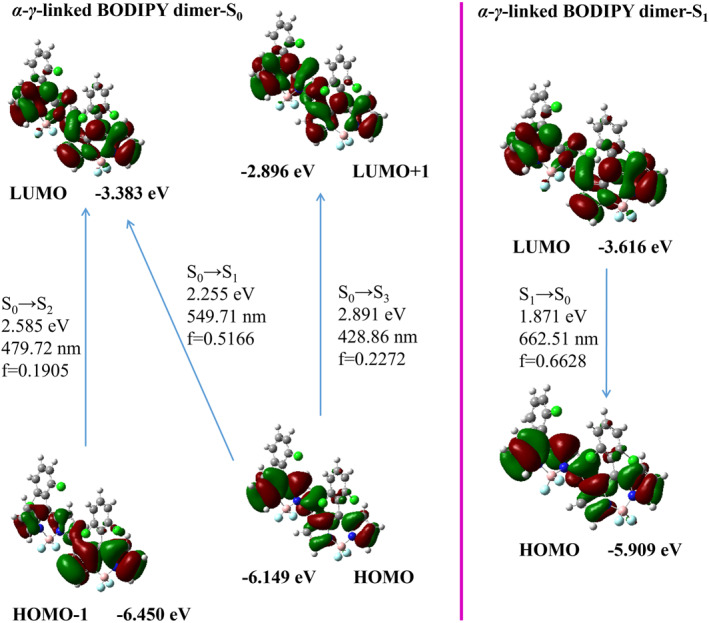
Frontier molecular orbitals of the optimized S_0_ state and the S_1_ state geometries for the *α*‐*γ*‐linked BODIPY dimer.

The *α*‐*γ*‐linked BODIPY dimer exhibited a main absorption band HOMO→LUMO under S_0_→S_1_ excitation with long wavelength absorption and moderate oscillator strength (549.71 nm, *f* = 0.5166). After vibrational relaxation, the S_1_→S_0_ transition from LUMO to HOMO showed long wavelength emission and the moderate oscillator strength (662.51 nm, *f* = 0.6628) in Figure 10. Therefore, the locations of the connection between two BODIPY individuals play a crucial role in modulating both the frontier molecular orbitals and the oscillator strength of their first singlet excited state. This further regulates the absorption and emission wavelengths as well as fluorescence intensity of these dyes.

In addition, the excitation energies of BODIPY dimers were overestimated using the TD‐DFT method, resulting in a lack of agreement between the theoretical and experimental values. However, the transition between energy levels can be analyzed with the help of frontier molecular orbitals, and the relationship between quantum yield and the ability of electron transition can be qualitatively analyzed.

### 3.4 The inactivation process of BODIPY dimers without installing blocking groups

In the *C*hole&*C*ele map, the distribution of holes and electrons is effectively described by the Gaussian function. The contour map of holes and electrons is presented as an ellipse, extremely smooth, which allows the type of electron excitation to be determined more clearly and intuitively. In the map, green represents the distribution of electrons, while blue represents the distribution of holes. The *D*_he_ index is designed to measure the distance between the centroid of the hole and the electron and *t* index is designed to measure the separation degree of the hole and electron in the‐transfer direction. The EDD serves as a crucial method for analyzing electronic structures, where the density disparity between the S_1_ and T_1_ states is quantified by subtracting the electron density in the S_1_ state from that in the T_1_ state. Within the EDD map, regions exhibiting an increase in electron density in the T_1_ state relative to the S_1_ state are denoted by green, while those showing a decrease are represented by blue, thereby distinctly delineating the trajectory of electron migration between the two electronic states.

**FIGURE 11:**
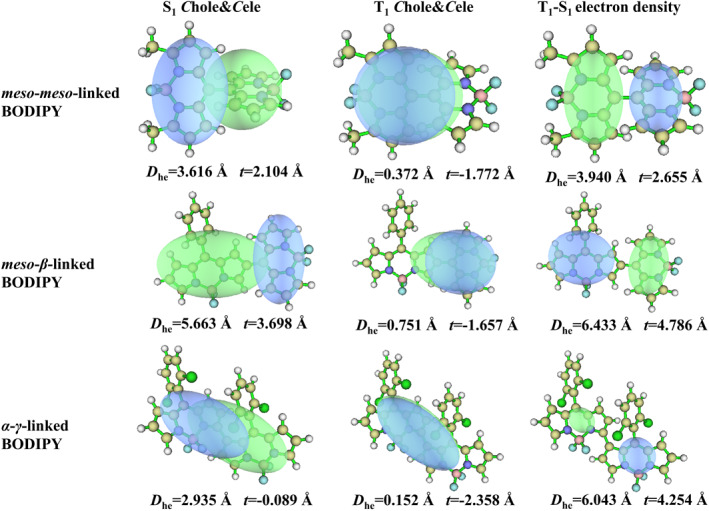
*C*hole&*C*ele map and electron density difference of *meso*‐*meso*‐linked BODIPY, *meso*‐*β*‐linked BODIPY and *α*‐*γ*‐linked BODIPY (isovalue = 0.0005).

For both *meso*‐*β*‐linked BODIPY and *meso*‐*meso*‐linked BODIPY, the centroid distance (*D*_he_) between the hole and electron in the S_1_ state is significantly large, and the separation degree of the hole and electron (*t*) is greater than 0 in Figure 11, which suggests a high degree of hole‐electron separation in the S_1_ state, characteristic of a charge‐transfer excitation (^1^CT) state. For *α*‐*γ*‐linked BODIPY, the S_1_ state exhibitted the *t*‐value of −0.089 Å, which is close to 0, but with the *D*_he_ of 2.931 Å, also classifying it as a charge‐transfer excitation state. In the T_1_ state of *meso*‐*β*‐linked BODIPY, *meso*‐*meso*‐linked BODIPY, and *α*‐*γ*‐linked BODIPY, there is a notable decrease in the centroid distance (*D*_he_) between the hole and electron, accompanied by a drop in the *t*‐value. These characteristics indicate that the T_1_ state is characteristic of a local excitation (^3^LE) state. The EDD maps for the T_1_‐S_1_ states of *meso*‐*β*‐linked BODIPY, *meso*‐*meso*‐linked BODIPY, and *α*‐*γ*‐linked BODIPY indicated that a substantial charge transfer occurs during the transition process. By integrating these EDD maps with the *C*hole&*C*ele maps, it is evident that the transition from the S_1_ to the T_1_ state involves a complex charge reorganization process, transitioning from ^1^CT state to ^3^LE state. This transition significantlyenhanced the change in orbital angular momentum, thereby promoting electron spin flipping and facilitating ISC. Subsequently, this led to the attainment of the T_1_ state and the energy transfer that generates singlet oxygen. This intricate process is in alignment with the SOCT‐ISC mechanism.

**FIGURE 12:**
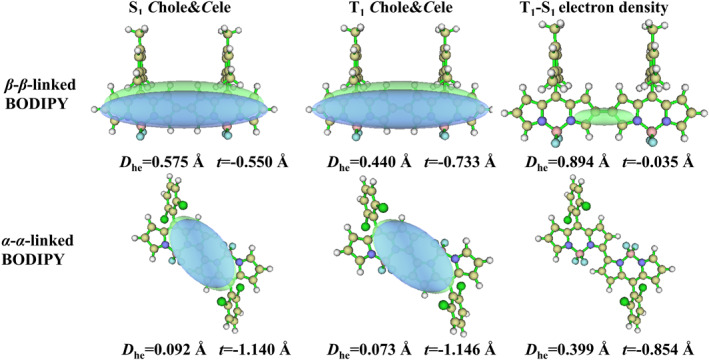
*C*hole&*C*ele map and electron density difference in *β*‐*β*‐linked BODIPY and *α*‐*α*‐linked BODIPY dimers (isovalue = 0.0005).

From the perspective of *C*hole&*C*ele maps for the S_1_ and T_1_ states in Figure 12, both *β*‐*β*‐linked BODIPY and *α*‐*α*‐linked BODIPY molecules displayed smaller *D*_he_ values and *t* values less than zero. This indicated a high degree of overlap between electrons and holes, characterizing them as local excitation states. Analysis of EDD maps revealed a negligible charge transfer within the *β*‐*β*‐linked and *α*‐*α*‐linked BODIPY molecules during the transition from the S_1_ to the T_1_ state. This observation strongly suggestted that the occurrence of a charge transfer recombination process transitioning from the S_1_ to the T_1_ state is highly improbable. Moreover, the absence of charge transfer hindered the spin inversion of S_1_ electrons through ISC to the T_1_ state, rendering these molecules unable to generate singlet oxygen.

**FIGURE 13:**
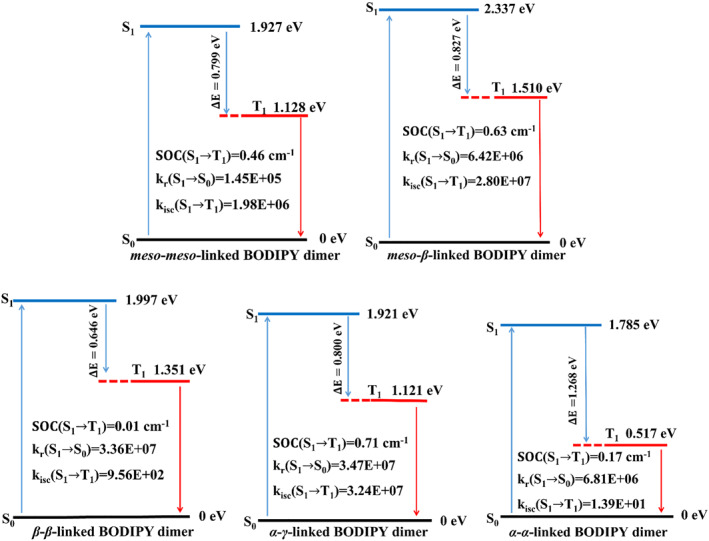
Calculated adiabatic energy, SOC value of S_1_ →T_1_, fluorescence radiative decay rate k_*r*_, ISC rates k_isc_. Notes: k_*r* (i0→f)_ = $\frac{64\;{\pi \;}^{4}}{h^{4}\;c^{3}}{\left|\mu \right|}^{2}\sum_{a}\;{\left(\nu_{\text{if}}+\sum_{j}a_{j}\nu_{j}\right)}^{3}\prod_{j}\frac{S_{j}^{a_{i}}}{a_{j}!}e^{{-S}_{j}}$ ^[65]^, *μ* is the electric transition dipole moment between the two states, *h* is the Planck constant, *c* is the speed of light in vacuum, *S*_*j*_ is the Huang‐Rhys factor for the *j*‐th mode, *a* is the number of vibrational quantums. k_isc_ = $\frac{2\pi }{h}{\langle S_{n}\left|{\hat{H}}_{SO}\right|T_{m}\rangle }^{2}\times$ [FCWD],^[66]^ $\langle S_{n}\left|{\hat{H}}_{SO}\right|T_{m}\rangle$ is a rotational orbit coupling matrix element between the S_*n*_ state and the T_*m*_ state, [FCWD] is Franck–Condon weighted density of states. ISC, intersystem crossing; SOC, spin‐orbit coupling.

Adiabatic energies for the optimized states, the energy gap for S_1_→T_1_, the energy gap for T_1_→S_0_, the SOC coefficient between S_1_ and T_1_, the fluorescence radiative decay rate k_*r*_, and the ISC rates k_isc_ are collected in Figure 13. A superior Type‐II photosensitizer should also have a large energy gap between T_1_ and S_0_ so that there is enough energy to sensitize ^3^O_2_ to ^1^O_2_, a small energy gap between T_1_ and S_1_ and a large SOC coefficient so that it can increase the ISC rate between S_1_ and T_1_. The *meso*‐*β*‐linked BODIPY dimer, *meso*‐*meso*‐linked BODIPY dimer, and *α*‐γ‐linked BODIPY dimer showed large energy gaps T_1_→S_0_ from 1.121 to 1.510 eV, small energy gaps S_1_→T_1_ from 0.800 to 0.827 eV and large SOC coefficients from 0.46 cm^−1^ to 0.71 cm^−1^, resulting in higher singlet oxygen response. The *β*‐*β*‐linked BODIPY dimer had a very small SOC coefficient (0.01 cm^−1^) between S_1_ state and T_1_ state, which indicated that *β*‐*β*‐linked BODIPY dimer did not produce effective ISC from S_1_ state to T_1_ state. In addition, the *α*‐*α*‐linked BODIPY dimer has a very low ISC rate and lacks the ability to sensitize triplet oxygen to singlet oxygen, which may be due to the relatively narrow energy gap of 0.517 eV between T_1_ and S_0_, the wide energy gap of 1.268 eV between S_1_ and T_1_, and the low SOC coefficient of 0.17 cm^−1^. The *β*‐*β*‐linked BODIPY dimer and the *α*‐*α*‐linked BODIPY dimer are not ideal photosensitizers, but rather superior fluorescent dyes.

The ISC rates (k_isc_) of S_1_→T_1_ for the *β*‐*β*‐linked and *α*‐*α*‐linked BODIPY dimers are 9.56 × 10^2^ and 1.39 × 10^1^, respectively, which are significantly lower than their the fluorescence radiative decay rates k_*r*_ (10^6^–10^7^). The triplet excited state of the *meso*‐*β*‐linked BODIPY dimer, *meso*‐*meso*‐linked BODIPY dimer, and *α*‐*γ*‐linked BODIPY dimer can be effectively generated, as evidenced by their k_isc_ of S_1_→T_1_, which falled within the range of approximately 10^6^–10^7^. We have discovered that the *α*‐*γ*‐linked BODIPY dimer exhibits fluorescence and ISC rates of the same order of magnitude, rendering it suitable as a photosensitizer for both therapeutic and imaging purposes.

We explored the potential energy profile of the C‐C bond rotation between two BODIPY units as they rotate in the S_0_ and S_1_ states of the BODIPY dimer. This is illustrated in Figure S1 and summarized in Table S3. The smoothness of the S_1_ state potential energy surface (PES) from 30° to 150° suggests that structural relaxation involving BODIPY unit rotation is achieved in the *meso*‐*β*‐linked BODIPY dimer, *meso*‐*meso*‐linked BODIPY dimer and *α*‐*γ*‐linked BODIPY dimer. The PES of S_1_ state from 30° to 150° of *β*‐*β*‐linked BODIPY dimer has a relatively high excited state energy barrier. But the PES of S_1_ state from 0° to 30° and from 160° to 180° of the *β*‐*β*‐linked BODIPY dimer is smoother, which indicates that *β*‐*β*‐linked BODIPY dimer can realize internal conversion within a small region. The PES of S_1_ state from 0° to 120° for the *α*‐*α*‐linked BODIPY dimers has a relatively high excited state energy barrier. However, the PES becomes smoother from 130° to 180°, indicating that the *α*‐*α*‐linked BODIPY dimer can undergo internal conversion in a small range. The *β*‐*β*‐linked BODIPY dimer and *α*‐*α*‐linked BODIPY dimer demonstrated low rotational energy barriers within a restricted range of local rotational angles, enabling rotation to access the nonradiative transition pathway and facilitating internal conversion. This implies that the excited states of these two dyes primarily return to the ground state through fluorescence and internal conversion, with minimal capability to undergo ISC to the triplet state. We cannot ignore the internal conversion efficiency of BODIPY dimers without installing blocking groups due to the local rotation of the C‐C bond of the two BODIPY units. With further molecular design, we expect to minimize internal conversion and improve fluorescence and ISC rates.

## 4 CONCLUSIONS

In a systematic study, the various optical properties and singlet oxygen quantum yields of a set of BODIPY dimers without any blocking groups installed were compared. We found that the different connection locations between two BODIPY units significantly affected the conjugation between BODIPY units, charge transfer strength and the SOC coefficient. Combining *C*hole&*C*ele map with EDD maps, it is revealed that *meso*‐*β*‐linked BODIPY, *meso*‐*meso*‐linked BODIPY and *α*‐*γ*‐linked BODIPY all exhibited marked charge transfer during the S_1_→T_1_ transition, corresponding to a transition from ^1^CT to ^3^LE state driven by SOCT‐ISC mechanism. The BODIPY dimers linked through *meso*‐*β* and *meso*‐*meso* bonds have large SOC coefficients for ISC, high T_1_ energy level to sensitive ^3^O_2_ to ^1^O_2_ and weak conjugation between the two BODIPY units in S_1_ state for low fluorescence quantum yield. The *α*‐*γ*‐linked BODIPY dimer with a large SOC coefficient, a higher T_1_ energy level, and relatively strong conjugation between two BODIPY units, can promote ISC processes and generate significant singlet oxygen, relatively long wavelength and pronounced fluorescence, which will serve as both therapeutic and imaging agents. In the *β*‐*β*‐linked BODIPY and *α*‐*α*‐linked BODIPY molecules, the occurrence of a charge transfer recombination process during the S_1_→T_1_ transition is unlikely, which is unfavorable for the ISC of S_1_→T_1_ state. In the T_1_ state, the planar arrangement of two BODIPY units in both *α*‐*α*‐linked and *β*‐*β*‐linked BODIPY dimers obstructed the ISC, resulting in low SOC values and consequently hindering the production of singlet oxygen. The *β*‐*β*‐linked BODIPY dimer and *α*‐*α*‐linked BODIPY dimer displayed high degree of conjugation between the two BODIPY units in the first excited state, resulting in relatively long and strong fluorescence. Our results may contribute to the design of BODIPY dimers without blocking groups, as well as the development of high‐performance dual‐functioning photosensitizers as therapeutic and imaging agents.

## CONFLICT OF INTEREST STATEMENT

The authors declare no conflicts of interest.

## ETHICS STATEMENT

Not applicable.

## Supporting information

Supporting Information S1

## Data Availability

Research data are not shared.

## References

[1] B. Ran , Y. Yuan , W. Xia , M. Li , Q. Yao , Z. Wang , L. Wang , X. Li , Y. Xu , X. Peng , Chem. Sci. 2020, 12, 1054. 34163871 10.1039/d0sc04889e PMC8179032

[2] M. Li , J. Xia , R. Tian , J. Wang , J. Fan , J. Du , S. Long , X. Song , J. W. Foley , X. Peng , J. Am. Chem. Soc. 2018, 140, 14851. 30362735 10.1021/jacs.8b08658

[3] M. Li , Y. Shao , J. H. Kim , Z. Pu , X. Zhao , H. Huang , T. Xiong , Y. Kang , G. Li , K. Shao , J. Fan , J. W. Foley , J. S. Kim , X. Peng , J. Am. Chem. Soc. 2020, 142, 5380. 32105455 10.1021/jacs.0c00734

[4] E. Avellanal‐Zaballa , A. Prieto‐Castaneda , C. Diaz‐Norambuena , J. Banuelos , A. R. Agarrabeitia , I. Garcia‐Moreno , S. de la Moya , M. J. Ortiz , Phys. Chem. Chem. Phys. 2021, 23, 11191. 33954326 10.1039/d1cp00991e

[5] S. Kaya , Y. A. Ismaiel , N. Kwon , G. Kim , J. L. Bila , J. Yoon , O. Seven , E. U. Akkaya , Dyes Pigm. 2021, 188, 109158.

[6] D. Ma , H. Bian , M. Gu , L. Wang , X. Chen , X. Peng , Coord. Chem. Rev. 2024, 505, 215677.

[7] X. F. Zhang , J. L. Zhu , J. Lumin. 2019, 212, 286.

[8] V. N. Nguyen , Y. Yan , J. Zhao , J. Yoon , Acc. Chem. Res. 2021, 54, 207. 33289536 10.1021/acs.accounts.0c00606

[9] J. Zou , Z. Yin , P. Wang , D. Chen , J. Shao , Q. Zhang , L. Sun , W. Huang , X. Dong , Chem. Sci. 2018, 9, 2188. 29719692 10.1039/c7sc04694d PMC5903368

[10] X. Zhang , Z. J. Wang , Y. Q. Hou , Y. X. Yan , J. Z. Zhao , B. Dick , J. Mater. Chem. C 2021, 9, 11944.

[11] J. Du , T. Shi , S. Long , P. Chen , W. Sun , J. Fan , X. Peng , Coord. Chem. Rev. 2021, 427, 213604.

[12] W. Jiang , M. Liang , Q. Lei , G. Li , S. Wu , Cancers 2023, 15, 585. 36765543 10.3390/cancers15030585 PMC9913255

[13] J. Wang , Q. Gong , L. Jiao , E. Hao , Coord. Chem. Rev. 2023, 496, 215367.

[14] Y. Yan , A. A. Sukhanov , M. H. E. Bousquet , Q. Guan , J. Zhao , V. K. Voronkova , D. Escudero , A. Barbon , Y. Xing , G. G. Gurzadyan , D. Jacquemin , J. Phys. Chem. B 2021, 125, 6280. 34077214 10.1021/acs.jpcb.1c03189

[15] S. Gao , S. Yu , Y. M. Zhang , A. H. Wu , S. H. Zhang , G. G. Wei , H. Wang , Z. Y. Xiao , W. Lu , Adv. Funct. Mater. 2021, 31, 2008356.

[16] Y. Liu , M. Gu , Q. Ding , Z. Zhang , W. Gong , Y. Yuan , X. Miao , H. Ma , X. Hong , W. Hu , Y. Xiao , Angew. Chem. Int. Ed. Engl. 2023, 62, e202214875. 36545827 10.1002/anie.202214875 PMC9880658

[17] L. A. Ortiz‐Rodriguez , S. J. Hoehn , A. Loredo , L. Wang , H. Xiao , C. E. Crespo‐Hernandez , J. Am. Chem. Soc. 2021, 143, 2676. 33587618 10.1021/jacs.0c13203 PMC7985834

[18] J. Deckers , T. Cardeynaels , S. Doria , N. Tumanov , A. Lapini , A. Ethirajan , M. Ameloot , J. Wouters , M. Di Donato , B. Champagne , W. Maes , J. Mater. Chem. C 2022, 10, 9344.

[19] R. Prieto‐Montero , A. Diaz Andres , A. Prieto‐Castaneda , A. Tabero , A. Longarte , A. R. Agarrabeitia , A. Villanueva , M. J. Ortiz , R. Montero , D. Casanova , V. Martinez‐Martinez , J. Mater. Chem. B 2022, 11, 169. 36484323 10.1039/d2tb01515c

[20] D. Wang , X. Wang , S. Zhou , P. Gu , X. Zhu , C. Wang , Q. Zhang , Coord. Chem. Rev. 2023, 482, 215074.

[21] H. B. Cheng , X. Cao , S. Zhang , K. Zhang , Y. Cheng , J. Wang , J. Zhao , L. Zhou , X. J. Liang , J. Yoon , Adv. Mater. 2023, 35, 2207546. 10.1002/adma.202207546 36398522

[22] H. Ning , Y. Yang , C. Lv , D. Zhou , S. Long , W. Sun , J. Du , J. Fan , X. Peng , Nano Res. 2023, 16, 12294.

[23] J. Zhao , K. Xu , W. Yang , Z. Wang , F. Zhong , Chem. Soc. Rev. 2015, 44, 8904. 26465741 10.1039/c5cs00364d

[24] X. Zhao , J. Liu , J. Fan , H. Chao , X. Peng , Chem. Soc. Rev. 2021, 50, 4185. 33527104 10.1039/d0cs00173b

[25] L. Zhang , Z. Huang , D. Dai , Y. Xiao , K. Lei , S. Tan , J. Cheng , Y. Xu , J. Liu , X. Qian , Org. Lett. 2016, 18, 5664. 27750427 10.1021/acs.orglett.6b02902

[26] Z. Wang , M. Ivanov , Y. Gao , L. Bussotti , P. Foggi , H. Zhang , N. Russo , B. Dick , J. Zhao , M. Di Donato , G. Mazzone , L. Luo , M. Fedin , Chem. Eur J. 2020, 26, 1091. 31743947 10.1002/chem.201904306

[27] K. X. Teng , W. K. Chen , L. Y. Niu , W. H. Fang , G. Cui , Q. Z. Yang , Angew. Chem. Int. Ed. Engl. 2021, 60, 19912. 34227724 10.1002/anie.202106748

[28] L. Li , G. Yuan , Q. Qi , C. Lv , J. Liang , H. Li , L. Cao , X. Zhang , S. Wang , Y. Cheng , H. He , J. Mater. Chem. B 2022, 10, 3550. 35420087 10.1039/d1tb02598h

[29] S. Qi , N. Kwon , Y. Yim , N. Van‐Nghia , J. Yoon , Chem. Sci. 2020, 11, 6479. 34094113 10.1039/d0sc01171a PMC8152625

[30] Y. Tang , X. Wang , G. Zhu , Z. Liu , X. M. Chen , H. K. Bisoyi , X. Chen , X. Chen , Y. Xu , J. Li , Q. Li , Small 2023, 19, e2205440. 36285777 10.1002/smll.202205440

[31] A. A. Pakhomov , A. S. Belova , A. G. Khchoyan , Y. N. Kononevich , D. S. Ionov , M. A. Maksimova , A. Y. Frolova , M. V. Alfimov , V. I. Martynov , A. M. Muzafarov , Molecules 2022, 27, 9060. 36558192 10.3390/molecules27249060 PMC9780792

[32] X. Zheng , L. Zhang , M. Ju , L. Liu , C. Ma , Y. Huang , B. Wang , W. Ding , X. Luan , B. Shen , ACS Appl. Mater. Interfaces 2022, 14, 46262. 36197147 10.1021/acsami.2c12781

[33] Y. Dong , A. Elmali , J. Zhao , B. Dick , A. Karatay , Chem. Phys. Chem. 2020, 21, 1388. 32391942 10.1002/cphc.202000300 PMC7383670

[34] V. N. Nguyen , Y. Yim , S. Kim , B. Ryu , K. M. K. Swamy , G. Kim , N. Kwon , C. Y. Kim , S. Park , J. Yoon , Angew. Chem. Int. Ed. Engl. 2020, 59, 8957. 32125064 10.1002/anie.202002843

[35] S. Radunz , S. Wedepohl , M. Rohr , M. Calderon , H. R. Tschiche , U. Resch‐Genger , J. Med. Chem. 2020, 63, 1699. 31967820 10.1021/acs.jmedchem.9b01873

[36] S. Li , Y. Chen , Y. Wu , S. Yao , H. Yuan , Y. Tan , F. Qi , W. He , Z. Guo , Chemistry 2022, 28, e202202680. 36170107 10.1002/chem.202202680

[37] Q. Gong , Q. Wu , X. Guo , W. Li , L. Wang , E. Hao , L. Jiao , Org. Lett. 2021, 23, 7220. 34463517 10.1021/acs.orglett.1c02622

[38] J. Deckers , T. Cardeynaels , H. Penxten , A. Ethirajan , M. Ameloot , M. Kruk , B. Champagne , W. Maes , Chem. Eur J. 2020, 26, 15212. 32584436 10.1002/chem.202002549

[39] H. Ito , H. Sakai , Y. Suzuki , J. Kawamata , T. Hasobe , Chem. Eur J. 2020, 26, 316. 31815329 10.1002/chem.201904282

[40] Y. Cakmak , S. Kolemen , S. Duman , Y. Dede , Y. Dolen , B. Kilic , Z. Kostereli , L. T. Yildirim , A. L. Dogan , D. Guc , E. U. Akkaya , Angew. Chem. Int. Ed. 2011, 50, 11937. 10.1002/anie.201105736 22012892

[41] B. Ventura , G. Marconi , M. Bröring , R. Krüger , L. Flamigni , New J. Chem. 2009, 33, 428.

[42] Z. S. Li , Y. Liu , X. F. Hou , Z. H. Xu , C. H. Liu , F. Z. Zhang , Z. G. Xie , J. Lumin. 2019, 212, 306.

[43] T. Ozdemir , J. L. Bila , F. Sozmen , L. T. Yildirim , E. U. Akkaya , Org. Lett. 2016, 18, 4821. 27712079 10.1021/acs.orglett.6b02418

[44] J. F. Cao , T. C. Zhang , W. Sun , Dyes Pigm. 2022, 208, 110197.

[45] D. Wang , Q. Wu , X. Zhang , W. Wang , E. Hao , L. Jiao , Org. Lett. 2020, 22, 7694. 32946245 10.1021/acs.orglett.0c02895

[46] X. F. Zhang , Dyes Pigm. 2017, 146, 491.

[47] X. F. Zhang , X. Yang , B. Xu , Phys. Chem. Chem. Phys. 2017, 19, 24792. 28868533 10.1039/c7cp02645e

[48] Y. Hayashi , S. Yamaguchi , W. Y. Cha , D. Kim , H. Shinokubo , Org. Lett. 2011, 13, 2992. 21591621 10.1021/ol200799u

[49] A. Austin , G. A. Petersson , M. J. Frisch , F. J. Dobek , G. Scalmani , K. Throssell , J. Chem. Theor. Comput. 2012, 8, 4989. 10.1021/ct300778e 26593191

[50] A. V. Marenich , C. J. Cramer , D. G. Truhlar , J. Phys. Chem. B 2009, 113, 6378. 19366259 10.1021/jp810292n

[51] M. J. Frisch , G. W. Trucks , H. B. Schlegel , G. E. Scuseria , M. A. Robb , J. R. Cheeseman , G. Scalmani , V. Barone , G. A. Petersson , H. Nakatsuji , X. Li , M. Caricato , A. V. Marenich , J. Bloino , B. G. Janesko , R. Gomperts , B. Mennucci , H. P. Hratchian , J. V. Ortiz , A. F. Izmaylov , J. L. Sonnenberg , D. Williams‐Young , F. Ding , F. Lipparini , F. Egidi , J. Goings , B. Peng , A. Petrone , T. Henderson , D. Ranasinghe , V. G. Zakrzewski , J. Gao , N. Rega , G. Zheng , W. Liang , M. Hada , M. Ehara , K. Toyota , R. Fukuda , J. Hasegawa , M. Ishida , T. Nakajima , Y. Honda , O. Kitao , H. Nakai , T. Vreven , K. Throssell , J. A. Montgomery, Jr. , J. E. Peralta , F. Ogliaro , M. J. Bearpark , J. J. Heyd , E. N. Brothers , K. N. Kudin , V. N. Staroverov , T. A. Keith , R. Kobayashi , J. Normand , K. Raghavachari , A. P. Rendell , J. C. Burant , S. S. Iyengar , J. Tomasi , M. Cossi , J. M. Millam , M. Klene , C. Adamo , R. Cammi , J. W. Ochterski , R. L. Martin , K. Morokuma , O. Farkas , J. B. Foresman , D. J. Fox , Gaussian, Inc., Wallingford CT 2016.

[52] T. Lu , F. Chen , J. Comput. Chem. 2012, 33, 580e592. 22162017 10.1002/jcc.22885

[53] Z. Y. Liu , T. Lu , Q. X. Chen , Carbon 2020, 165, 461.

[54] W. Liu , G. Hong , D. Dai , L. Li , M. Dolg , Theor. Chem. Acc. 1997, 96, 75.

[55] Y. Zhang , B. Suo , Z. Wang , N. Zhang , Z. Li , Y. Lei , W. Zou , J. Gao , D. Peng , Z. Pu , Y. Xiao , Q. Sun , F. Wang , Y. Ma , X. Wang , Y. Guo , W. Liu , J. Chem. Phys. 2020, 152, 064113. 32061235 10.1063/1.5143173

[56] W. Liu , F. Wang , L. Li , J. Theor. Comput. Chem. 2011, 02, 257.02.

[57] W. J. Liu , F. Wang , L. Li , Vol. 5, World Scientific Publishing 2004, p. 257.

[58] Z. Li , B. Suo , Y. Zhang , Y. Xiao , W. Liu , Mol. Phys. 2013, 111, 3741.

[59] Z. Li , Y. Xiao , W. Liu , J. Chem. Phys. 2012, 137, 154114. 23083155 10.1063/1.4758987

[60] Z. Li , Y. Xiao , W. Liu , J. Chem. Phys. 2014, 141, 054111. 25106574 10.1063/1.4891567

[61] Y. L. Niu , W. Q. Li , Q. Peng , H. Geng , Y. P. Yi , L. J. Wang , G. J. Nan , D. Wang , Z. G. Shuai , Mol. Phys. 2018, 116, 1078.

[62] Q. Peng , Y. Yi , Z. Shuai , J. Shao , J. Am. Chem. Soc. 2007, 129, 9333. 17622142 10.1021/ja067946e

[63] Y. Niu , Q. Peng , Z. Shuai , Sci. China, Ser. B: Chem. 2008, 51, 1153.

[64] Z. G. Shuai , Chin. J. Chem. 2020, 38, 1223.

[65] Q. Peng , Y. Yi , Z. Shuai , J. Shao , J. Am. Chem. Soc. 2007, 129, 9333. 17622142 10.1021/ja067946e

[66] E. Runge , E. K. U. Gross , Phys. Rev. Lett. 1984, 52, 997.

