# Supplementary material for: The controllable of BODIPY dimers without installing blocking groups as both fluorescence and singlet oxygen generators
Source: Smart Mol. 2024 Jul 21;3(1):e20240023. doi: 10.1002/smo.20240023 (PMC12117891; doi:10.1002/smo.20240023)
Supplement: Supplementary file 1 — Supporting Information S1 [file SMO2-3-e20240023-s001.docx]

Supporting Information

The Controllable of BODIPY Dimers without Installing Blocking Groups as Both Fluorescence and Singlet Oxygen Generators

*Jianfang Cao^1^, Tianci Zhang^1^ , Xinyu Chen**^1^, Xue Ma^1^, and Jiangli Fan ^2^**

^1^ School of Chemical Engineering, Ocean and Life Sciences, Dalian University of Technology, Panjin Campus, Panjin, 124221, China.

^2^ State Key Laboratory of Fine Chemicals, Dalian University of Technology, No. 2 Linggong Road, High-tech District, Dalian 116024, China.

Correspondence

Jiangli Fan

E-mail: [fanjl@dlut.edu.cn](mailto:fanjl@dlut.edu.cn)；Tel.: +86-411-849-86327

Table S1 the energy separations for the absorption ΔE_abs_(nm) and corresponding oscillator strengths of the low-lying electronically excited states for *meso*-*β*-linked BODIPY dimer, *meso*-*meso*-linked BODIPY dimer，*β*-*β*-linked BODIPY dimer，*α*-*α*-linked BODIPY dimer and *α*-*γ*-linked BODIPY dimer dyes.

| Compound | Transition | Energy  (eV) | Maximum absorption wavelength  (nm) | Oscillator strengths f | Composition | CI |
| --- | --- | --- | --- | --- | --- | --- |
| *meso*-*β* | S_0_→S_1_ | 2.5433 | 487.50 | 0.0724 | HOMO→LUMO | 0.69097 |
|  | S_0_→S_2_ | 2.6595 | 466.20 | 0.7436 | HOMO-1→LUMO | 0.67544 |
|  | S_0_→S_3_ | 2.9779 | 416.35 | 0.4034 | HOMO→LUMO+1 | 0.67384 |
|  | S_0_→S_4_ | 3.0824 | 402.23 | 0.0730 | HOMO-1→LUMO+1 | 0.67427 |
| *meso*-*meso* | S_0_→S_1_ | 2.1450 | 578.00 | 0.0476 | HOMO→LUMO | 0.64647 |
|  | S_0_→S_2_ | 2.5776 | 481.00 | 0.1540 | HOMO→LUMO+1 | 0.47674 |
|  | S_0_→S_3_ | 2.7982 | 443.08 | 0.3437 | HOMO-1→LUMO | 0.52470 |
|  | S_0_→S_4_ | 3.0100 | 411.91 | 0.4853 | HOMO-1→LUMO+1 | 0.60836 |
| *β*-*β* | S_0_→S_1_ | 2.2035 | 562.67 | 0.9014 | HOMO→LUMO | 0.70335 |
|  | S_0_→S_2_ | 2.5864 | 479.37 | 0.0101 | HOMO→LUMO+1 | 0.67922 |
|  | S_0_→S_3_ | 2.8963 | 428.08 | 0.0001 | HOMO-2→LUMO | 0.61854 |
|  | S_0_→S_4_ | 2.8975 | 427.90 | 0.0000 | HOMO-3→LUMO | 0.61736 |
|  | S_0_→S_5_ | 3.0376 | 408.17 | 0.0087 | HOMO-1→LUMO | 0.59953 |
| *α*-*α* | S_0_→S_1_ | 2.0970 | 591.25 | 1.0664 | HOMO→LUMO | 0.70751 |
|  | S_0_→S_2_ | 2.6829 | 462.12 | 0.0025 | HOMO→LUMO+1 | 0.54330 |
|  | S_0_→S_3_ | 3.0088 | 412.07 | 0.1436 | HOMO-1→LUMO | 0.51434 |
|  | S_0_→S_4_ | 3.1826 | 389.57 | 0.0000 | HOMO-2→LUMO | 0.68277 |
| *α-γ* | S_0_→S_1_ | 2.2554 | 549.71 | 0.5166 | HOMO→LUMO | 0.69236 |
|  | S_0_→S_2_ | 2.5845 | 479.72 | 0.1905 | HOMO-1→LUMO | 0.61512 |
|  | S_0_→S_3_ | 2.8910 | 428.86 | 0.2272 | HOMO→LUMO+1 | 0.56993 |
|  | S_0_→S_4_ | 3.0309 | 409.07 | 0.2594 | HOMO-1→LUMO+1 | 0.64655 |

Table S2 the energy separations for the emission ΔE_em_(nm) and corresponding oscillator strengths of the low-lying electronically excited states for *meso*-*β*-linked BODIPY dimer, *meso*-*meso*-linked BODIPY dimer，*β*-*β*-linked BODIPY dimer，*α*-*α*-linked BODIPY dimer and *α*-*γ*-linked BODIPY dimer dyes.

| Compound | Transition | Energy  (eV) | Maximum emission wavelength  (nm) | Oscillator strengths f | Composition | CI |
| --- | --- | --- | --- | --- | --- | --- |
| *meso*-*β* | S_1_→S_0_ | 2.1845 | 567.58 | 0.0514 | HOMO←LUMO | 0.70159 |
|  | S_2_→S_0_ | 2.6018 | 476.52 | 0.8321 | HOMO-1←LUMO | 0.68255 |
|  | S_3_→S_0_ | 2.8963 | 428.07 | 0.3764 | HOMO←LUMO+1 | 0.69093 |
|  | S_4_→S_0_ | 3.2220 | 384.80 | 0.0852 | HOMO-1←LUMO | 0.45906 |
| *meso*-*meso* | S_1_→S_0_ | 1.7532 | 707.17 | 0.0274 | HOMO←LUMO | 0.68935 |
|  | S_2_→S_0_ | 2.7242 | 455.12 | 0.4725 | HOMO←LUMO+1 | 0.65997 |
|  | S_3_→S_0_ | 2.7915 | 444.14 | 0.3114 | HOMO-1←LUMO | 0.66302 |
|  | S_4_→S_0_ | 3.1179 | 397.66 | 0.0810 | HOMO-1←LUMO | 0.52601 |
| *β*-*β* | S_1_→S_0_ | 2.0051 | 618.33 | 0.8714 | HOMO←LUMO | 0.70506 |
|  | S_2_→S_0_ | 2.4071 | 515.07 | 0.0119 | HOMO←LUMO+1 | 0.69037 |
|  | S_3_→S_0_ | 2.8856 | 429.66 | 0.0003 | HOMO-2←LUMO | 0.62642 |
|  | S_4_→S_0_ | 2.8868 | 429.49 | 0.0000 | HOMO-3←LUMO | 0.62564 |
| *α*-*α* | S_1_→S_0_ | 1.8852 | 657.68 | 1.3083 | HOMO←LUMO | 0.71059 |
|  | S_2_→S_0_ | 2.6330 | 470.89 | 0.0023 | HOMO←LUMO+1 | 0.57759 |
|  | S_3_→S_0_ | 2.9820 | 415.77 | 0.0485 | HOMO-1←LUMO | 0.53426 |
|  | S_4_→S_0_ | 3.0476 | 406.83 | 0.0000 | HOMO-2←LUMO | 0.69367 |
|  | S_5_→S_0_ | 3.0506 | 406.42 | 0.0038 | HOMO-3←LUMO | 0.68361 |
|  | S_6_→S_0_ | 3.2230 | 384.69 | 0.0685 | HOMO-4←LUMO | 0.68805 |
| α*-*γ | S_1_→S_0_ | 1.8714 | 662.51 | 0.6628 | HOMO←LUMO | 0.70070 |
|  | S_2_→S_0_ | 2.4799 | 499.95 | 0.3225 | HOMO-1←LUMO | 0.59168 |
|  | S_3_→S_0_ | 2.8230 | 439.19 | 0.1008 | HOMO←LUMO+1 | 0.57654 |
|  | S_4_→S_0_ | 2.9251 | 423.86 | 0.0056 | HOMO-2←LUMO | 0.65549 |
|  | S_5_→S_0_ | 3.0770 | 402.94 | 0.0404 | HOMO-4←LUMO | 0.60248 |
|  | S_6_→S_0_ | 3.1508 | 393.50 | 0.0015 | HOMO-3←LUMO | 0.58426 |

Figure S1. Energy levels of the S_0_ (black) and S_1_ (red) states of rotation around chemical bonds (ϕ) between the two BODIPY units of BODIPY dimers.

Table S3. Energy barrier E_a_ and energy gap E_gap_ between the S_0_ and S_1_ states of rotation around chemical bonds (ϕ_1_) between the two BODIPY units in the S_0_ and S_1_ states of BODIPY dimers.

|  | *meso*-*meso* | *meso*-*β* | *α*-*γ* | *β*-*β* | *α*-*α* |
| --- | --- | --- | --- | --- | --- |
| E_a_  (kcal•mol^-1^) | 5.936 | 5.889 | 6.085 | 1.382 | 2.491 |
| E_gap_  (kcal•mol^-1^) | 47.688 | 55.832 | 49.027 | 52.737 | 49.381 |

The excited-state barriers calculated in this work are slightly overestimated, due to the fact that the molecule was kept rigid except for rotation about a particular bond. But this strategy allows us to separate the effect of a single rotation from other rotations. ^[1-3]^ It was found that allowing the molecule to relax along the remaining internal coordinates made little difference to the qualitative conclusions.

1. Imran M，Sukhanov A A，Wang Z，Karatay A，Zhao, J.; Mahmood, Z, et al. Electronic Coupling and Spin-Orbit Charge-Transfer Intersystem Crossing in Phenothiazine-Perylene Compact Electron Donor/ Acceptor Dyads. J. Phys. Chem. C 2019, 123 (12), 7010-7024.
2. Cao J, Sun W, Fan J, Insights into bishemicyanines with long emission wavelengths and high sensitivity in viscous environments. Chin. Chem. Lett. 2020, 31 (6), 1402-1405.
3. Cao X, Tolbert R W, McHale J L, Edwards W D, Theoretical Study of Solvent Effects on the Intramolecular Charge Transfer of a Hemicyanine Dye. J. Phys. Chem. A 1998, 102 (17), 2739-2748.

XYZ coordinates (angstrom) and SCF Energies (a.u.)

Note: upper case letters before the atomic coordinates indicate the atomic symbol of the atoms

involved in the calculations.

*α*-*α*-linked BODIPY in the S_0_ state

Energy = -3660.854565

C 7.22109700 1.11100200 1.63416600

C 6.08342000 0.35305500 1.40995800

C 5.33177900 0.47840100 0.24285200

C 5.76783200 1.40382100 -0.70416700

C 6.90159000 2.17509500 -0.50601800

C 7.62345800 2.02168500 0.66837600

C 4.12058300 -0.34098200 0.02004600

C 2.88294200 0.14006100 0.43809100

C 4.22532000 -1.55316000 -0.64089200

N 1.71904100 -0.58136300 0.26111700

C 0.68985700 0.21467300 0.62993900

C 1.19045200 1.46145400 1.06310800

C 2.55991200 1.40797800 0.95964800

C 5.33622700 -2.20157400 -1.22265000

C 4.85956400 -3.35740000 -1.81027300

C 3.47413400 -3.39366100 -1.57274600

N 3.09963300 -2.31672300 -0.88173800

B 1.69680300 -2.03649100 -0.29333100

F 0.72791300 -2.17555300 -1.27381600

F 1.46570700 -2.94150000 0.74675600

N -1.71902800 0.58121900 0.26109000

C -2.88296400 -0.14014400 0.43806700

C -4.12058400 0.34097700 0.02005100

C -4.22526500 1.55318700 -0.64083700

N -3.09954000 2.31669600 -0.88168200

B -1.69669700 2.03634300 -0.29335700

C -0.68988100 -0.21486100 0.62991200

C -1.19053200 -1.46162400 1.06306800

C -2.55998900 -1.40809400 0.95957800

C -5.33615200 2.20169600 -1.22253100

C -4.85943800 3.35752100 -1.81011400

C -3.47399800 3.39368700 -1.57263000

C -5.33182500 -0.47834300 0.24285000

C -6.08341800 -0.35302000 1.40999000

C -7.22114200 -1.11090000 1.63418500

C -7.62359700 -2.02149300 0.66835100

C -6.90178200 -2.17487500 -0.50607900

C -5.76797600 -1.40366900 -0.70421600

F -0.72785200 2.17531100 -1.27390100

F -1.46544300 2.94132600 0.74671500

H 7.78306800 0.98916300 2.55266000

H 7.21352300 2.88579800 -1.26218700

H 0.58801400 2.27770100 1.42617900

H 3.27293200 2.18175800 1.20501600

H 6.35175200 -1.83251500 -1.20690300

H 5.42155300 -4.10021000 -2.35711300

H 2.75320400 -4.14314700 -1.86840700

H -0.58813800 -2.27789400 1.42615700

H -3.27304600 -2.18184400 1.20493800

H -6.35169800 1.83269700 -1.20676800

H -5.42139900 4.10039000 -2.35690200

H -2.75303200 4.14314100 -1.86828300

H -7.78307600 -0.98907900 2.55270400

H -7.21379000 -2.88550700 -1.26228400

H 8.51259500 2.62056100 0.83369500

Cl 4.86489300 1.60226600 -2.17630300

Cl 5.58147400 -0.78875900 2.62188300

Cl -5.58135900 0.78869200 2.62196500

Cl -4.86510500 -1.60207800 -2.17639900

H -8.51274700 -2.62034700 0.83368000

*α*-*α*-linked BODIPY in the S_1_ state

Energy = -3660.788980

C 6.99593900 1.53636000 1.68844500

C 5.89909400 0.71585000 1.48235600

C 5.34599200 0.51679900 0.21660800

C 5.94581600 1.18991700 -0.84900100

C 7.04449800 2.01561300 -0.67365400

C 7.56485800 2.18283900 0.60117300

C 4.17807100 -0.36350000 0.02022700

C 2.88762100 0.13207800 0.25184000

C 4.34805100 -1.65954300 -0.44350600

N 1.75535200 -0.63011700 0.12130400

C 0.67178700 0.20566400 0.29772300

C 1.16511200 1.52455100 0.55572300

C 2.52460400 1.47094700 0.55337200

C 5.51138600 -2.37354200 -0.82270300

C 5.08883200 -3.60896000 -1.27244100

C 3.68464300 -3.63295500 -1.15428200

N 3.24900400 -2.46849000 -0.66441500

B 1.80070400 -2.13636300 -0.25408400

F 0.93436800 -2.41290200 -1.30421200

F 1.46610100 -2.92659900 0.85515100

N -1.75533400 0.63004300 0.12119800

C -2.88762700 -0.13210500 0.25175600

C -4.17806400 0.36349800 0.02010000

C -4.34799600 1.65945300 -0.44387600

N -3.24891100 2.46830000 -0.66496500

B -1.80066000 2.13623800 -0.25441000

C -0.67179600 -0.20577600 0.29766700

C -1.16516800 -1.52462600 0.55570800

C -2.52466700 -1.47097200 0.55335300

C -5.51128800 2.37336900 -0.82336000

C -5.08867000 3.60864600 -1.27342900

C -3.68448600 3.63262800 -1.15521200

C -5.34602500 -0.51671000 0.21668000

C -5.89929300 -0.71523300 1.48243400

C -6.99619000 -1.53562800 1.68870800

C -7.56497800 -2.18253200 0.60161900

C -7.04444900 -2.01583100 -0.67320900

C -5.94572700 -1.19022600 -0.84874000

F -0.93417800 2.41264200 -1.30443700

F -1.46623900 2.92664000 0.85477900

H 7.39931100 1.66504700 2.68596300

H 7.48328600 2.52455800 -1.52375000

H 0.54998600 2.38199800 0.76783500

H 3.22044700 2.27775800 0.73049100

H 6.52037500 -1.99009500 -0.77294200

H 5.69738000 -4.41814600 -1.64978900

H 2.99485300 -4.43072200 -1.39308100

H -0.55011000 -2.38211800 0.76784200

H -3.22052400 -2.27776700 0.73049300

H -6.52028800 1.98995500 -0.77355900

H -5.69716900 4.41774100 -1.65105400

H -2.99466000 4.43030300 -1.39421800

H -7.39969200 -1.66391400 2.68622600

H -7.48314700 -2.52509400 -1.52316000

H 8.42430300 2.82773100 0.74961000

Cl 5.28508100 1.01017200 -2.44778600

Cl 5.19562000 -0.09919600 2.84889100

Cl -5.19590900 0.10028000 2.84873400

Cl -5.28482900 -1.01101600 -2.44751600

H -8.42445700 -2.82734300 0.75020500

*α*-*α*-linked BODIPY in the T_1_ state

Energy = -3660.835642

C -6.83420400 -1.96805600 1.44794300

C -5.76430000 -1.09537600 1.33344600

C -5.34487400 -0.57284300 0.10684800

C -6.06537000 -0.98223200 -1.01868700

C -7.14568100 -1.84678000 -0.93807700

C -7.52439800 -2.33671100 0.30281900

C -4.20651100 0.35933700 0.01160900

C -2.88695700 -0.10867300 0.09965900

C -4.40578000 1.72165400 -0.18934700

N -1.80086900 0.66711300 0.05731000

C -0.64204200 -0.19844800 0.10699500

C -1.16319200 -1.56314400 0.19777600

C -2.49315800 -1.51239400 0.21721500

C -5.61188300 2.47593000 -0.34027600

C -5.23974500 3.78818100 -0.53897700

C -3.83833900 3.83118000 -0.50584300

N -3.35043200 2.57646400 -0.29629500

B -1.87658400 2.20566500 -0.13659000

F -1.15636700 2.59089800 -1.27083100

F -1.35361400 2.87103900 0.98339400

N 1.80090900 -0.66718500 0.05761300

C 2.88699300 0.10860600 0.09987600

C 4.20654600 -0.35938200 0.01173300

C 4.40581200 -1.72169000 -0.18930800

N 3.35046800 -2.57655200 -0.29588200

B 1.87663400 -2.20576800 -0.13602200

C 0.64207700 0.19838700 0.10698200

C 1.16321800 1.56310600 0.19742400

C 2.49317900 1.51235500 0.21710000

C 5.61189800 -2.47589600 -0.34078700

C 5.23974500 -3.78815100 -0.53943100

C 3.83835800 -3.83122800 -0.50571900

C 5.34487700 0.57284100 0.10691600

C 5.76418600 1.09556900 1.33347500

C 6.83400900 1.96835100 1.44793000

C 7.52425800 2.33690100 0.30280700

C 7.14566400 1.84677400 -0.93804900

C 6.06541000 0.98214900 -1.01862400

F 1.15631300 -2.59123200 -1.27012000

F 1.35378400 -2.87093100 0.98414100

H -7.12505400 -2.35002100 2.41936200

H -7.67830300 -2.13822700 -1.83566100

H -0.55450000 -2.44485900 0.28234000

H -3.18980900 -2.33410400 0.29636600

H -6.60877200 2.06127400 -0.30475300

H -5.88771400 4.63875600 -0.69363900

H -3.17242800 4.67463100 -0.61546700

H 0.55452100 2.44484900 0.28162900

H 3.18981800 2.33408900 0.29612100

H 6.60878100 -2.06119400 -0.30563000

H 5.88769700 -4.63868300 -0.69440500

H 3.17244700 -4.67470800 -0.61512400

H 7.12475600 2.35047500 2.41931900

H 7.67832500 2.13814600 -1.83563500

H -8.36621500 -3.01677300 0.37796200

Cl -5.58754700 -0.40517200 -2.59012000

Cl -4.91117900 -0.63795800 2.78127200

Cl 4.91101100 0.63827900 2.78130900

Cl 5.58770600 0.40491300 -2.59003100

H 8.36601700 3.01703900 0.37791900

*α*-*γ*-linked BODIPY in the S_0_ state

Energy = -3660.860225

C -3.06080900 -0.52584200 0.01102600

C -4.35984200 -0.78204500 0.43226500

C -2.74430800 0.67279700 -0.62857900

N -3.72538700 1.62710000 -0.82380300

C -3.15267200 2.73292000 -1.30267100

C -1.77605500 2.54631700 -1.43076500

C -1.50279900 1.24666900 -1.02371300

C -4.88635800 -1.88768600 1.13582000

C -6.23308500 -1.64251700 1.32009400

C -6.50317600 -0.39712800 0.72829400

N -5.38838000 0.11287000 0.20396200

B -5.24143200 1.43728200 -0.57281900

F -5.91863900 1.35883100 -1.79001900

F -5.74113600 2.50033800 0.17053700

C 3.17764600 0.44923200 -0.03673300

C 3.39343500 1.51338100 0.82694500

C 1.93490100 0.24893500 -0.62366100

N 0.87513300 1.10732700 -0.39159600

C -0.20103400 0.60599700 -1.02464800

C 0.15814400 -0.58174300 -1.69599300

C 1.49151400 -0.80584800 -1.44730700

C 4.51967100 1.86597900 1.59899100

C 4.16568600 2.98264100 2.33380900

C 2.83875700 3.29156000 1.99069000

N 2.38025500 2.41278200 1.09780300

B 1.02698300 2.46924600 0.34916100

F 1.07094700 3.50063400 -0.59504500

F -0.00509400 2.68707200 1.24135000

C -2.03309800 -1.55047600 0.30052700

C 4.27759300 -0.49770000 -0.32333500

C -1.17096900 -1.42732600 1.39006800

C -0.20403400 -2.37885900 1.66634800

C -0.09538600 -3.49104300 0.84618500

C -0.95220600 -3.66130200 -0.23095200

C -1.90989500 -2.69500200 -0.48611800

C 5.09618200 -0.33225600 -1.43998500

C 6.12991700 -1.20839300 -1.72795600

C 6.35736600 -2.28274100 -0.88082300

C 5.56540200 -2.48041600 0.24044600

C 4.53945900 -1.58770000 0.50494000

H -3.74295200 3.60746400 -1.53761400

H -1.06368600 3.27149000 -1.79051700

H -4.31834800 -2.74801900 1.45926600

H -6.95546700 -2.26886500 1.82246500

H -7.44086100 0.13758800 0.66609800

H -0.50901100 -1.16932900 -2.30637300

H 2.10355900 -1.62493100 -1.79645100

H 5.46058200 1.33469700 1.60735700

H 4.77312800 3.52514400 3.04332400

H 2.21508600 4.10072900 2.34455900

H 0.45006400 -2.25064000 2.51978700

H -0.87384600 -4.53059700 -0.87302400

H 6.74863900 -1.04972800 -2.60331300

H 5.74148700 -3.31809100 0.90485500

Cl 4.82071700 1.01680400 -2.50197400

Cl 3.55070600 -1.84293200 1.91210900

Cl -1.31274000 -0.05410100 2.43899400

Cl -2.94984400 -2.89752000 -1.86574700

H 7.16392600 -2.97500000 -1.09692700

H 0.66394500 -4.23767100 1.05209600

*α*-*γ*-linked BODIPY in the S_1_ state

Energy = -3660.789568

C -3.09607500 -0.54509700 -0.00488900

C -4.44010400 -0.86302600 0.21192800

C -2.75946800 0.71706200 -0.54635100

N -3.72455400 1.65555800 -0.74147300

C -3.13648400 2.85821000 -1.00918200

C -1.77416800 2.73574900 -0.97459500

C -1.48950300 1.36462500 -0.74200700

C -5.02607100 -2.00875300 0.79605900

C -6.39917600 -1.81015300 0.78901900

C -6.62690700 -0.55364300 0.21754200

N -5.45542000 0.00863900 -0.12811600

B -5.24952500 1.41606700 -0.69978600

F -5.77787300 1.50899300 -1.99274700

F -5.86129700 2.37632500 0.10735400

C 3.23129500 0.44034200 -0.13529900

C 3.58011000 1.55053100 0.62138400

C 1.92310400 0.29837700 -0.59684600

N 0.94383000 1.23857100 -0.35447300

C -0.23541100 0.72605600 -0.79074300

C 0.02109100 -0.56244100 -1.38248300

C 1.34988300 -0.81551200 -1.27329900

C 4.79724000 1.88661700 1.26956100

C 4.57640100 3.07055700 1.93487700

C 3.23535700 3.44410100 1.67888900

N 2.64880300 2.53652800 0.90172200

B 1.22438300 2.62132700 0.29903500

F 1.20774400 3.61627500 -0.68089700

F 0.30730800 2.90920700 1.29047800

C -2.07101500 -1.49468100 0.46124600

C 4.22130200 -0.62197300 -0.40406100

C -1.28630500 -1.21597400 1.58949700

C -0.30447100 -2.08035600 2.04159400

C -0.10431100 -3.28190900 1.37937400

C -0.88224000 -3.61831100 0.28154400

C -1.84736100 -2.72963800 -0.15973900

C 4.93191300 -0.66242200 -1.60367700

C 5.86079700 -1.65348700 -1.87495900

C 6.09061900 -2.63725300 -0.92454500

C 5.40294200 -2.63413500 0.28014900

C 4.48031700 -1.63020300 0.52470700

H -3.74173800 3.72743400 -1.22044100

H -1.05607100 3.51173500 -1.18303600

H -4.48412300 -2.86513700 1.17004100

H -7.16219800 -2.47993200 1.15877300

H -7.56033200 -0.03288000 0.05611700

H -0.72611500 -1.14955400 -1.88999600

H 1.89731800 -1.67900400 -1.62100400

H 5.70074000 1.29501200 1.23913900

H 5.27283400 3.62781500 2.54426600

H 2.69753900 4.31820000 2.02017200

H 0.28587100 -1.82063600 2.91216900

H -0.72952700 -4.55580100 -0.23996700

H 6.39677300 -1.65377700 -2.81670500

H 5.57821500 -3.40409400 1.02219500

Cl 4.64550700 0.57034300 -2.79612700

Cl 3.61069700 -1.64146400 2.03063900

Cl -1.56506800 0.24580400 2.48254900

Cl -2.74010800 -3.14251500 -1.59572600

H 6.81535000 -3.41865900 -1.12606400

H 0.66236900 -3.96637800 1.72592400

*α*-*γ*-linked BODIPY in the T_1_ state

Energy = -3660.819011

C 3.15192900 -0.47965200 0.05567500

C 4.52280600 -0.73096300 -0.08907900

C 2.74171100 0.77904100 0.52018200

N 3.64889600 1.75467300 0.74330500

C 2.99437600 2.96638200 0.85205100

C 1.65367200 2.80679000 0.69769200

C 1.41135100 1.40761700 0.58038200

C 5.18725300 -1.86983400 -0.61937100

C 6.54334300 -1.61150700 -0.56013100

C 6.69175800 -0.33219800 -0.00867800

N 5.47697700 0.18694300 0.27115000

B 5.17981300 1.58456800 0.82212800

F 5.60446400 1.69999700 2.14992500

F 5.80819800 2.56744900 0.05818800

C -3.30662800 0.39010800 0.11620000

C -3.70327600 1.48921600 -0.63457100

C -1.97252000 0.28910700 0.52119600

N -1.03907800 1.23991000 0.25362300

C 0.19457900 0.74033600 0.64566900

C -0.02437800 -0.55122500 1.23739000

C -1.34797000 -0.81954500 1.18855500

C -4.95840300 1.79977000 -1.23384100

C -4.79516200 2.99155400 -1.90222500

C -3.45947200 3.39491500 -1.70615200

N -2.81514900 2.48756700 -0.94956200

B -1.36817500 2.59385700 -0.43031900

F -1.29853000 3.63419800 0.50519200

F -0.49703300 2.84899300 -1.47896700

C 2.21699600 -1.50841000 -0.44486600

C -4.26126700 -0.69350900 0.42085100

C 1.51310300 -1.32729200 -1.64060600

C 0.63770800 -2.27866100 -2.13634900

C 0.46807100 -3.46735100 -1.44338600

C 1.16884700 -3.70438900 -0.27098500

C 2.02789200 -2.72961300 0.20803000

C -5.16374400 -0.59823000 1.48137100

C -6.07090100 -1.60398000 1.77347500

C -6.08347200 -2.74701300 0.98804800

C -5.20257900 -2.88541000 -0.07442200

C -4.30861400 -1.86165200 -0.34264600

H 3.55825000 3.86577600 1.05239900

H 0.90774900 3.57836700 0.78360600

H 4.69929300 -2.75692400 -0.99551700

H 7.35007500 -2.25483100 -0.87989800

H 7.59295800 0.23121000 0.18692100

H 0.74407200 -1.13137200 1.71920000

H -1.86971100 -1.68509400 1.56923400

H -5.84609700 1.18804500 -1.16376000

H -5.53249500 3.53208500 -2.47759600

H -2.95219000 4.27751100 -2.06921000

H 0.10315400 -2.09283400 -3.06039200

H 1.04145500 -4.63174100 0.27487500

H -6.75523100 -1.49419200 2.60643500

H -5.21105300 -3.77656000 -0.69083200

Cl -5.14591300 0.82393400 2.48311400

Cl -3.21176300 -2.04241200 -1.68140000

Cl 1.74312600 0.13055000 -2.55703400

Cl 2.84152500 -3.02661700 1.71920100

H -6.78884400 -3.54131800 1.20734200

H -0.21557700 -4.21879700 -1.82325300

*β*-*β*-linked linked BODIPY in the S_0_ state

Energy = -2058.602020

C 5.30057800 -0.79799600 0.04296600

N 5.31100700 -2.18135000 0.10873200

B 4.05740000 -3.08376500 0.14524900

N 2.83329200 -2.13793600 0.07707100

C 2.89188000 -0.75479500 0.01556900

C 4.10918500 -0.08068100 -0.00232900

C 6.63562200 -0.34850400 0.03201200

C 7.44130300 -1.47299400 0.09152600

C 6.58447700 -2.58266400 0.13766100

C 1.55233000 -2.49648100 0.07437500

C 0.72311000 -1.35571800 0.01024000

C 1.57879600 -0.26004600 -0.02584700

C 4.12971900 1.39856500 -0.07107100

C 4.13147400 2.03017500 -1.32101300

C 4.13932100 3.42088300 -1.36181000

C 4.14552600 4.18863700 -0.20042100

C 4.13962600 3.53210600 1.02649700

C 4.13246000 2.14274700 1.11446100

C 4.18346500 5.68656800 -0.27298400

C 4.12087800 1.46454500 2.45305100

C 4.11865500 1.23118700 -2.59116500

F 4.02547000 -3.82244700 1.32723300

F 4.05254000 -3.95300800 -0.94427300

C -2.89186700 -0.75480800 -0.01538700

N -2.83327100 -2.13795300 -0.07678600

B -4.05737100 -3.08377900 -0.14517100

N -5.31098100 -2.18136100 -0.10887800

C -5.30056100 -0.79800400 -0.04314900

C -4.10917400 -0.08069100 0.00230000

C -1.57878700 -0.26005100 0.02607400

C -0.72309500 -1.35572100 -0.00993800

C -1.55230700 -2.49648900 -0.07409100

C -6.58444700 -2.58267700 -0.13794500

C -7.44128000 -1.47300800 -0.09188800

C -6.63560800 -0.34851500 -0.03232600

C -4.12971600 1.39855700 0.07100300

C -4.13131300 2.03020400 1.32097300

C -4.13920600 3.42087000 1.36175000

C -4.14559100 4.18863000 0.20031800

C -4.13986500 3.53210200 -1.02655700

C -4.13267500 2.14269800 -1.11450500

C -4.18336400 5.68655200 0.27315700

C -4.12139600 1.46451000 -2.45310500

C -4.11831200 1.23120500 2.59111600

F -4.05271100 -3.95303100 0.94434200

F -4.02522600 -3.82244900 -1.32715800

H 8.52076700 -1.51200600 0.10133800

H 4.13814000 3.91718300 -2.32886900

H 4.13805100 4.11556300 1.94362100

H 5.21422500 6.04505100 -0.37205400

H 3.76477900 6.14158900 0.62764900

H 3.62741700 6.05860300 -1.13717700

H 4.99497200 0.81855200 2.57977400

H 3.23762100 0.82903200 2.56976500

H 4.12072800 2.19675300 3.26228000

H 4.13676900 1.88569900 -3.46425700

H 3.22534400 0.60239300 -2.65659000

H 4.98243100 0.56199700 -2.64973500

H -8.52074300 -1.51202300 -0.10180000

H -4.13792800 3.91720100 2.32879800

H -4.13845000 4.11553500 -1.94369100

H -3.77273900 6.14170000 -0.63109000

H -3.61978900 6.05872700 1.13243500

H -5.21328200 6.04473900 0.38153900

H -4.12048800 2.19673200 -3.26232200

H -4.99602700 0.81927300 -2.58000200

H -3.23866800 0.82824100 -2.56967600

H -4.98200700 0.56191300 2.64973200

H -4.13644400 1.88570700 3.46421500

H -3.22492500 0.60251300 2.65647400

H 6.94116700 0.68679700 -0.01506900

H 6.83448900 -3.63338900 0.18958000

H -6.94115900 0.68678600 0.01470400

H -6.83445000 -3.63340300 -0.18988400

H 1.30975500 0.78478400 -0.08029400

H 1.26544900 -3.53803900 0.12023900

H -1.30975300 0.78478100 0.08049500

H -1.26542000 -3.53804700 -0.11991100

*β*-*β*-linked BODIPY in the S_1_ state

Energy = -2058.528635

C -5.28591200 -0.82090600 -0.01662300

N -5.26799900 -2.21107200 -0.05754500

B -4.00991800 -3.09295600 -0.10684000

N -2.79697800 -2.12611000 -0.05377100

C -2.87341400 -0.72285700 -0.02012700

C -4.11875700 -0.06832300 -0.00100200

C -6.63734600 -0.40378800 0.00960500

C -7.41519400 -1.54821900 -0.01497400

C -6.53518300 -2.64218900 -0.05662100

C -1.52436400 -2.47796900 -0.04618200

C -0.70444000 -1.31660500 -0.00917000

C -1.58689800 -0.21177100 0.00260900

C -4.16767600 1.40928400 0.04368600

C -4.15660300 2.06484700 1.28173900

C -4.19078700 3.45601000 1.30161800

C -4.23455500 4.20418100 0.12840000

C -4.24021600 3.52740600 -1.08793900

C -4.20850000 2.13723700 -1.15220300

C -4.30086800 5.70228500 0.17552800

C -4.21454200 1.43502600 -2.47833500

C -4.10653000 1.28552400 2.56311300

F -3.96945600 -3.83369600 -1.28815900

F -3.96960500 -3.96239600 0.98204300

C 2.87325500 -0.72296900 0.02019700

N 2.79676200 -2.12621900 0.05389700

B 4.00965200 -3.09312400 0.10694400

N 5.26777300 -2.21131200 0.05750500

C 5.28575100 -0.82115100 0.01648300

C 4.11863400 -0.06850200 0.00092800

C 1.58676000 -0.21183000 -0.00246400

C 0.70425700 -1.31663300 0.00937200

C 1.52413100 -2.47802500 0.04640500

C 6.53493700 -2.64249600 0.05649500

C 7.41499700 -1.54857400 0.01468100

C 6.63720400 -0.40410400 -0.00991100

C 4.16767900 1.40910300 -0.04381100

C 4.15580100 2.06462700 -1.28182500

C 4.19024300 3.45584100 -1.30173200

C 4.23506900 4.20397200 -0.12858700

C 4.24146700 3.52719200 1.08779200

C 4.20953400 2.13708100 1.15207900

C 4.30186000 5.70209000 -0.17518200

C 4.21651500 1.43486100 2.47820300

C 4.10469100 1.28532600 -2.56316600

F 3.96921700 -3.96263600 -0.98188100

F 3.96923300 -3.83379100 1.28831200

H -8.49371300 -1.61343300 -0.00429300

H -4.18038600 3.96837400 2.26035900

H -4.26732600 4.09591800 -2.01420100

H -5.34060300 6.04402600 0.23105000

H -3.85864900 6.15066400 -0.71736500

H -3.78246300 6.09815100 1.05227900

H -5.09543900 0.79413600 -2.58392300

H -3.33817400 0.78919800 -2.59113500

H -4.21650800 2.15152700 -3.30172600

H -4.10236300 1.95225000 3.42729700

H -3.21047500 0.65896500 2.61300900

H -4.96695900 0.61545000 2.65452600

H 8.49351300 -1.61384100 0.00389900

H 4.17928000 3.96822500 -2.26044600

H 4.26942900 4.09569000 2.01405100

H 5.34239200 6.04397900 -0.21158400

H 3.84361100 6.15062300 0.70958000

H 3.79922200 6.09757800 -1.06113500

H 4.21844800 2.15135800 3.30159700

H 5.09781600 0.79445800 2.58340700

H 3.34056500 0.78853600 2.59137100

H 4.96485900 0.61499000 -2.65510700

H 4.10020200 1.95205900 -3.42734500

H 3.20840800 0.65905100 -2.61252700

H -6.96598800 0.62469600 0.04471300

H -6.76319300 -3.69879200 -0.08531800

H 6.96589700 0.62435900 -0.04513200

H 6.76289500 -3.69910800 0.08524000

H -1.32320600 0.83437000 0.03340200

H -1.22331800 -3.51578700 -0.07322900

H 1.32310600 0.83432000 -0.03328200

H 1.22304400 -3.51582900 0.07351500

*β*-*β*-linked BODIPY in the T_1_ state

Energy = -2058.552374

C 5.28832200 -0.83666000 0.01995500

N 5.26493300 -2.21867100 0.06228200

B 4.00282900 -3.09141300 0.10610700

N 2.79670900 -2.11577800 0.05492000

C 2.88229700 -0.71640000 0.01918300

C 4.12430400 -0.07106400 0.00133900

C 6.64463100 -0.42248000 -0.00253100

C 7.41870700 -1.56759600 0.02635200

C 6.53712000 -2.65841700 0.06639900

C 1.51927700 -2.45586800 0.04770900

C 0.70870300 -1.27983200 0.00883300

C 1.58842500 -0.19418400 -0.00477900

C 4.18745100 1.40746500 -0.04381600

C 4.19082100 2.06361200 -1.28144000

C 4.23944100 3.45449200 -1.30224600

C 4.28387900 4.20265100 -0.12901900

C 4.27495600 3.52626900 1.08756000

C 4.22806300 2.13638800 1.15138700

C 4.36645800 5.70003000 -0.17609300

C 4.21711100 1.43504700 2.47804600

C 4.13939500 1.28469300 -2.56310300

F 3.95405900 -3.83815700 1.28417900

F 3.95662100 -3.95755700 -0.98636300

C -2.88215100 -0.71651500 -0.01914600

N -2.79650500 -2.11589000 -0.05493300

B -4.00257900 -3.09157800 -0.10618800

N -5.26472000 -2.21890100 -0.06231200

C -5.28817000 -0.83689600 -0.01988500

C -4.12418900 -0.07123800 -0.00124600

C -1.58829900 -0.19424700 0.00480800

C -0.70853300 -1.27985900 -0.00884600

C -1.51905900 -2.45592800 -0.04776100

C -6.53689000 -2.65870600 -0.06642900

C -7.41852400 -1.56792800 -0.02628900

C -6.64450000 -0.42278000 0.00266400

C -4.18745900 1.40728400 0.04396200

C -4.19011900 2.06341100 1.28150300

C -4.23901100 3.45437000 1.30232300

C -4.28440500 4.20245400 0.12918300

C -4.27610700 3.52601500 -1.08746300

C -4.22899100 2.13623200 -1.15129500

C -4.36745600 5.69982100 0.17570900

C -4.21878900 1.43481300 -2.47791900

C -4.13772000 1.28458400 2.56318500

F -3.95633200 -3.95779400 0.98622400

F -3.95376600 -3.83824500 -1.28430900

H 8.49703900 -1.63466100 0.01963900

H 4.24017800 3.96673400 -2.26112400

H 4.30248600 4.09498500 2.01369500

H 5.41010200 6.03086900 -0.22394000

H 3.92235700 6.15343100 0.71335100

H 3.85878200 6.10114500 -1.05673900

H 5.08291300 0.77416800 2.58522400

H 3.32635200 0.80900800 2.59016100

H 4.23395900 2.15173300 3.30116500

H 4.14918500 1.95106900 -3.42756500

H 3.23596100 0.66925900 -2.61871800

H 4.99157600 0.60338900 -2.64889700

H -8.49685400 -1.63504200 -0.01955600

H -4.23922400 3.96659700 2.26119800

H -4.30434900 4.09473900 -2.01358300

H -5.41170300 6.03083500 0.20585500

H -3.90850500 6.15356300 -0.70604600

H -3.87448200 6.10044500 1.06482800

H -4.23577600 2.15145800 -3.30106900

H -5.08483800 0.77418800 -2.58469400

H -3.32827300 0.80849100 -2.59036200

H -4.98949000 0.60281900 2.64938000

H -4.14750200 1.95099900 3.42761800

H -3.23392200 0.66965300 2.61845700

H 6.97524400 0.60526200 -0.03746600

H 6.75930300 -3.71572500 0.09732100

H -6.97516000 0.60494400 0.03768700

H -6.75902500 -3.71602200 -0.09741200

H 1.33596400 0.85470200 -0.03647100

H 1.20687200 -3.49020300 0.07616300

H -1.33588000 0.85464700 0.03653800

H -1.20661300 -3.49025000 -0.07627000

*meso*-*β*-linked BODIPY in the S_0_ state

Energy = -1591.885786

C -2.83511900 4.06485600 -0.14286900

C -2.58410900 2.73151200 -0.43083000

C -3.11660600 1.72584500 0.38086800

C -3.89436900 2.07542400 1.48791300

C -4.12745200 3.41126600 1.78097600

C -3.60260300 4.40758000 0.96512700

C -2.85567600 0.31334800 0.07313300

C -1.53233200 -0.13589400 -0.07378700

C -3.90621300 -0.58595400 -0.07501900

N -1.25000800 -1.45654600 -0.38153500

C 0.07045900 -1.61175700 -0.39833700

C 0.70159000 -0.38682600 -0.11251500

C -0.32067600 0.54218400 0.09333400

C -5.30591800 -0.38612000 -0.02943000

C -5.89909100 -1.60969300 -0.26695500

C -4.85890500 -2.53180500 -0.46808800

N -3.67788900 -1.92568700 -0.35433200

B -2.29758800 -2.57497300 -0.60035700

F -2.07910600 -3.61342700 0.30031100

F -2.21919900 -3.06127500 -1.90249800

C 2.13240200 -0.15943800 -0.04659100

C 2.69071000 0.98827800 -0.62717900

C 2.96539400 -1.08157300 0.60156500

N 4.04937500 1.23895800 -0.55574100

C 4.29989900 2.37637900 -1.21199200

C 3.11138200 2.90186000 -1.73156800

C 2.09855100 2.02619500 -1.37491300

C 2.65923600 -2.24736000 1.33370200

C 3.85793200 -2.76590300 1.79545600

C 4.86740600 -1.90308300 1.35330400

N 4.33655600 -0.90154300 0.64518100

B 5.10672600 0.30113900 0.06282700

F 5.81008700 0.95813900 1.07311900

F 6.00516000 -0.12412600 -0.91751300

H -2.43158000 4.83913000 -0.78747400

H -4.71868300 3.67493600 2.65204700

H 0.52173500 -2.56315100 -0.64073800

H -0.20821000 1.57384600 0.38852600

H -5.79664200 0.55923200 0.14766600

H -6.95477800 -1.83446600 -0.30588600

H -4.92092900 -3.58700400 -0.69632500

H 3.02279700 3.81067400 -2.30860800

H 4.00348200 -3.65342500 2.39365500

H -3.79124700 5.45190600 1.19309100

H 1.05208800 2.09906600 -1.62894900

H 1.66863400 -2.63711900 1.51401800

H 5.30820500 2.75775900 -1.29350200

H 5.93361400 -1.95607900 1.52417300

H -1.99776500 2.46120300 -1.30243400

H -4.29047900 1.29666200 2.13079300

*meso*-*β*-linked BODIPY in the S_1_ state

Energy = -1591.799915

C -3.05015600 4.02483400 -0.26131400

C -2.71121600 2.69885400 -0.49011500

C -3.25765000 1.67286400 0.29140000

C -4.15214100 2.01963400 1.31118700

C -4.48749300 3.34673200 1.54121800

C -3.93917900 4.35575700 0.75634000

C -2.89655200 0.27256200 0.04630500

C -1.53635900 -0.09913000 -0.03685000

C -3.88883600 -0.71926400 -0.10243700

N -1.17299500 -1.41992700 -0.28920600

C 0.15399600 -1.52411000 -0.23952100

C 0.71550800 -0.26699200 0.03155800

C -0.36954800 0.63660300 0.14968700

C -5.29184500 -0.62767400 -0.09812100

C -5.79245000 -1.91423300 -0.32112000

C -4.69951200 -2.75659000 -0.46633300

N -3.55660300 -2.04243400 -0.33384100

B -2.14896800 -2.58428400 -0.56120300

F -1.86697200 -3.64999900 0.30601700

F -1.99083500 -3.04180700 -1.87975300

C 2.11954900 -0.02592000 0.09217700

C 2.70285200 1.18637100 -0.34759300

C 2.98891700 -0.99776200 0.63488700

N 4.08242500 1.35295500 -0.38978400

C 4.33652100 2.56728000 -0.85617100

C 3.11896300 3.25390300 -1.12332800

C 2.10248300 2.39172100 -0.81565700

C 2.68795300 -2.15681100 1.40824900

C 3.88008000 -2.76733700 1.69587500

C 4.90181400 -1.95919900 1.13046200

N 4.36780300 -0.91118000 0.51505900

B 5.14232400 0.24556500 -0.15411700

F 6.13967100 0.70719400 0.68669300

F 5.67966500 -0.16096200 -1.36655900

H -2.62535000 4.80381100 -0.88739600

H -5.17564700 3.59487000 2.34365800

H 0.65374300 -2.45410900 -0.46764600

H -0.32512000 1.67477200 0.43352700

H -5.86114900 0.27855100 0.04215200

H -6.83037000 -2.21050800 -0.37644000

H -4.66698300 -3.81801600 -0.66458100

H 3.04304900 4.26058900 -1.50606900

H 4.04400600 -3.66609100 2.27127600

H -4.20339000 5.39319800 0.93596500

H 1.04372600 2.56794300 -0.90977200

H 1.70104100 -2.44839800 1.73160600

H 5.35241900 2.90714600 -1.00438800

H 5.97393300 -2.09219000 1.17701300

H -2.03412300 2.44461800 -1.29901500

H -4.56894400 1.23911500 1.93881200

*meso*-*β*-linked BODIPY in the T_1_ state

Energy = -1591.830282

C -2.87600700 4.06379700 -0.11314800

C -2.61340700 2.73393200 -0.40835700

C -3.14211200 1.71808200 0.39257400

C -3.92996700 2.05513000 1.49613400

C -4.17631600 3.38723000 1.79653500

C -3.65383000 4.39355800 0.99151400

C -2.86727000 0.30869600 0.07640000

C -1.54828600 -0.12991100 -0.06070200

C -3.92091800 -0.59621000 -0.09098700

N -1.26008300 -1.44389600 -0.38803500

C 0.06299100 -1.58516900 -0.41637000

C 0.69595700 -0.36660200 -0.09641000

C -0.32909600 0.54588600 0.13180400

C -5.31655400 -0.39888500 -0.06499200

C -5.90650300 -1.62489700 -0.32843200

C -4.86568200 -2.54053800 -0.52133900

N -3.68477800 -1.92910400 -0.37967400

B -2.29906400 -2.56940800 -0.59734800

F -2.08173600 -3.59338200 0.32503000

F -2.20037800 -3.08533100 -1.88915900

C 2.13443300 -0.14483300 -0.03869100

C 2.73199700 0.98324900 -0.63384800

C 2.99119100 -1.04431800 0.62574500

N 4.07299000 1.20936200 -0.58644500

C 4.34126500 2.36827200 -1.26672100

C 3.14288300 2.90651300 -1.76681000

C 2.12993300 2.06201100 -1.38835600

C 2.67479200 -2.23789800 1.37984300

C 3.86113300 -2.75367000 1.83815000

C 4.88500200 -1.90063800 1.39173100

N 4.34113700 -0.87568600 0.66361500

B 5.12369900 0.29196600 0.05152900

F 5.84199700 0.97465100 1.03975200

F 6.02703000 -0.16749600 -0.91424300

H -2.47388800 4.84548300 -0.74981600

H -4.77664300 3.64013600 2.66468500

H 0.51711000 -2.53340400 -0.66866700

H -0.22557100 1.57716600 0.43349000

H -5.81237400 0.54316900 0.11568100

H -6.96168700 -1.84861300 -0.38619900

H -4.91885100 -3.59372300 -0.75984500

H 3.06050000 3.81557600 -2.34459300

H 4.00438800 -3.63991600 2.43903900

H -3.85281800 5.43481000 1.22481400

H 1.07718800 2.14655800 -1.60901900

H 1.67800300 -2.61520000 1.55047500

H 5.35380700 2.73301500 -1.35879900

H 5.95127300 -1.95784500 1.55474700

H -2.01819700 2.47355500 -1.27704400

H -4.32624800 1.26899400 2.12991400

*meso*-*meso*-linked BODIPY in the S_0_ state

Energy = -1439.550736

C 1.10132800 0.00001700 0.00000800

C 1.79017900 -0.81861200 0.88938500

C 1.79018500 0.81864100 -0.88937000

N 3.17409700 -0.83804300 0.91228400

C 3.55707800 -1.68527800 1.86960000

C 1.32048500 -1.69900000 1.88755100

C 1.32049800 1.69901900 -1.88754800

C 2.43391100 2.24348800 -2.49973200

C 3.55709000 1.68532000 -1.86956200

N 3.17410300 0.83806400 -0.91226700

B 4.10023000 -0.00001800 -0.00001400

F 4.90465800 -0.83717400 -0.77034400

F 4.90475400 0.83708400 0.77027200

C -0.37562900 0.00001000 0.00000200

C -1.06114800 -1.11836600 -0.45877700

C -1.06116700 1.11837400 0.45878300

N -2.44575300 -1.15130500 -0.47398800

C -2.83335200 -2.33287700 -0.98203100

C -1.69141900 -3.09128500 -1.31033200

C -0.58359000 -2.33553500 -0.98729800

C -0.58362900 2.33554300 0.98732300

C -1.69147100 3.09128000 1.31034500

C -2.83339100 2.33285500 0.98203800

N -2.44577200 1.15129200 0.47399000

B -3.36338700 -0.00000600 -0.00002100

F -4.17143600 -0.43335300 1.05287800

F -4.17138300 0.43334600 -1.05296000

H 4.60549400 -1.85957800 2.06876200

H 0.28129100 -1.89219700 2.11004100

H 0.28130500 1.89221000 -2.11005100

H 2.45763700 2.95843700 -3.30900400

H 4.60550800 1.85962700 -2.06871100

H -1.71244700 -4.08342200 -1.73782500

H -1.71251600 4.08341500 1.73784100

H 0.45733000 -2.59911000 -1.10546400

H 0.45728600 2.59913100 1.10550200

C 2.43389500 -2.24339900 2.49980400

H 2.45761600 -2.95830200 3.30911700

C -4.25369600 2.71935400 1.14596300

H -4.77568200 2.71635900 0.18485900

H -4.77893500 2.01691000 1.79915400

H -4.32236000 3.71786400 1.57770400

C -4.25365000 -2.71940000 -1.14595500

H -4.77561500 -2.71648400 -0.18483900

H -4.77892100 -2.01692500 -1.79908700

H -4.32229700 -3.71788300 -1.57776100

*meso*-*meso*-linked BODIPY in the S_1_ state

Energy = -1439.475417

C 1.09090000 -0.00508300 0.00393300

C 1.79067500 0.87920000 0.85002400

C 1.79085300 -0.89099100 -0.84031500

N 3.16758900 -0.90019300 -0.84884100

C 3.58276600 -1.82005200 -1.75838300

C 2.48157400 -2.41633600 -2.34995300

C 1.34024000 -1.83291200 -1.78002000

C 1.33961700 1.81572400 1.79487000

C 2.48068300 2.39756000 2.36696600

C 3.58213300 1.80606500 1.77106800

N 3.16732300 0.88993700 0.85759700

B 4.07530400 0.00460600 -0.00563200

F 4.88574000 0.79692400 -0.83937200

F 4.92395700 -0.76785700 0.80767300

C -0.37247000 -0.00259000 0.00177000

C -1.08208400 1.10407000 -0.45621400

C -1.08817300 -1.10604800 0.45785900

N -2.47503100 -1.15927900 0.43761900

C -2.85221400 -2.32586600 0.95634000

C -1.68487400 -3.08249500 1.32213300

C -0.59829400 -2.32532100 1.02262400

C -0.58520600 2.32144200 -1.01897600

C -1.66755400 3.08341200 -1.32154000

C -2.83929800 2.33165200 -0.95973100

N -2.46878700 1.16336800 -0.44006700

B -3.40923900 0.00398200 -0.00278900

F -4.19620800 0.39950000 1.06535500

F -4.19431200 -0.38830400 -1.07352000

H 2.51126200 -3.18419700 -3.10997500

H 2.51006900 3.16084900 3.13159000

H -1.71266300 -4.06788800 1.76327100

H -1.68970700 4.06899800 -1.76258400

H 0.30805000 -2.05352500 -2.00969800

H 0.30727700 2.03269000 2.02735600

H 4.63499100 1.97773800 1.93899100

H 4.63569100 -1.99034800 -1.92730500

H 0.46107200 2.54791100 -1.15539100

H 0.44658400 -2.55631300 1.16216400

C -4.23494200 2.73936400 -1.16949500

H -4.50469000 2.54860100 -2.21674200

H -4.93221600 2.19518500 -0.53518100

H -4.33721500 3.81429400 -1.00577000

C -4.25035200 -2.72739700 1.16139700

H -4.52424200 -2.53105300 2.20655700

H -4.94252300 -2.18295700 0.52169200

H -4.35619000 -3.80251800 1.00161500

*meso*-*meso*-linked BODIPY in the T_1_ state

Energy = -1439.505351

C -1.08932400 0.00020200 0.00013200

C -1.80568700 -1.00071800 0.68391800

C -1.80579100 1.00116400 -0.68348200

N -3.18387200 1.04955000 -0.65323900

C -3.58506500 2.06067500 -1.43889400

C -2.47455200 2.69983900 -1.99787600

C -1.35301100 2.02560300 -1.53965500

C -1.35276700 -2.02482500 1.54041800

C -2.47423100 -2.69893400 1.99901200

C -3.58482900 -2.06007100 1.43985100

N -3.18375600 -1.04919100 0.65382400

B -4.10795500 -0.00028400 -0.00034800

F -4.91499600 -0.59710100 -0.96930900

F -4.91707900 0.59566600 0.96736200

C 0.35228600 0.00013100 0.00002700

C 1.08721400 -1.13628100 -0.41448900

C 1.08745600 1.13639800 0.41448000

N 2.44269400 1.16945100 0.39576700

C 2.86065900 2.38004000 0.90821000

C 1.72187500 3.13051900 1.26579700

C 0.61599200 2.37416000 0.98461800

C 0.61545700 -2.37397200 -0.98455100

C 1.72116900 -3.13053400 -1.26584700

C 2.86013200 -2.38025400 -0.90840700

N 2.44244000 -1.16958300 -0.39591900

B 3.36511700 -0.00016000 -0.00009500

F 4.17307200 -0.36455000 1.07783200

F 4.17311500 0.36405600 -1.07804200

H -2.50494100 3.54293300 -2.67251700

H -2.50452000 -3.54174000 2.67401700

H 1.75018000 4.11968400 1.69966400

H 1.74924200 -4.11971000 -1.69970300

H -0.32051100 2.22336500 -1.78717300

H -0.32023400 -2.22238700 1.78796200

H -4.63639900 -2.26401700 1.58392300

H -4.63665900 2.26457900 -1.58285700

H -0.42157900 -2.61944500 -1.15274000

H -0.42098000 2.61981900 1.15293000

C 4.28296900 -2.74078800 -1.04409000

H 4.79756700 -2.05273500 -1.72242500

H 4.79755000 -2.67626700 -0.08029400

H 4.37888300 -3.75525500 -1.43086100

C 4.28357500 2.74036400 1.04362700

H 4.79838600 2.05182100 1.72129400

H 4.79777900 2.67645000 0.07958200

H 4.37971500 3.75457900 1.43100500
